# Supplementary material for: Tumor-specific CD8 T cell characterization in HR+ breast cancer reveals an impaired antitumoral response in patients with lymph node metastasis
Source: Cell Rep Med. 2025 Jul 28;6(8):102252. doi: 10.1016/j.xcrm.2025.102252 (PMC12432377; doi:10.1016/j.xcrm.2025.102252)
Supplement: Document S1. Figures S1–S7 and Tables S1–S5 [file mmc1.pdf]

Cell Reports Medicine, Volume *6*

Supplemental information

Tumor-specific CD8 T cell characterization in HR$^+$ breast cancer reveals an impaired antitumoral response in patients with lymph node metastasis

Mariana Pereira Pinho, Elie Antoun, Balraj Sandhar, Ting Shu, Fei Gao, Xiaobao Yang, Adam Bates, Lucia Cerundolo, Megat H.B.A. Hamid, David Maldonado-Perez, Renuka Teague, Eve Warner, Lucinda Winter, Nasullah Khalid Alham, Clare Verrill, Simon R. Lord, Timothy Rostron, Sally-Ann Clark, Craig Waugh, Paul Sopp, Chris Conlon, Ricardo A. Fernandes, Adrian L. Harris, Yanchun Peng, Asha Adwani, and Tao Dong

Table S1: Correlation between the presence of a detectable tumor-reactive CD8 T cell response and clinical characteristics of the patients. The p-value was calculated using Fisher's exact test for the contingency table (Preoperative treatment) and t-test and Mann-Whitney for normally distributed variables, or not, respectively. A patient was considered to have a detectable circulating tumor-reactive CD8 T cell response if the percentage of $CFSE^{low}CD25^{+}$ CD8 T cells after coculture with DCs loaded with tumor lysate was higher than the baseline proliferation with unloaded DCs. Related to Figure 1.

Characteristic	Patients with an undetectable circulating tumor-reactive T cell response (n = 7)	Patients with a detectable circulating tumor-reactive T cell response (n = 16)	p-value
Age - median (range)	65 (51-83)	69 (39-86)	0.6251
Tumor size (mm) - median (range)	27 (17.5-60)	36 (19-120)	0.3155
Preop treatment (Letrozole):			
Yes - n (%)	1 (14.3)	2 (12.5)	>0.999
No - n (%)	6 (85.7)	14 (87.5)	
Progesterone score - median (range)	2 (0-8)	6 (0-8)	0.2703
Tumor grade - median (range)	2 (1-3)	2.5 (2-3)	0.2597

Table S2: Breast cancer subtype and HLA typing of the established cancer cell lines. TNBC: Triple-negative breast cancer. HR^{+}: Hormone receptor positive. Related to STAR Methods and Figure 2.

Cell line	Subtype	HLA-A		HLA-B		HLA-C	
BT20	TNBC	24:02	24:03	15:01	38:01	03:03	12:03
HCC1937	TNBC	23:01	24:02	07:02	40:01	03:04	07:02
MCF-7	HR^{+}	02:01	02:01	18:01	44:02	05:01/03	05:01/03
MDA-MB-231	TNBC	02:01	02:17	40:02	41:01	02:02	17:01/02
SUM159PT	TNBC	02:01	24:02	15:01	51:01	03:03	15:02
MDA-MB-436	TNBC	01:01/04N	01:01/04N	08:01	08:01	07:01	07:01

Table S3: HLA typing of breast cancer patients. Related to Figure 2.

Patient ID	HLA-A		HLA-B		HLA-C	
Br1	02:01	24:02	07:02	55:01	03:03	07:02
Br4	01:01/04N	03:01	53:01:00	57:01:00	04:01	06:02
Br6	32:01:00	32:01:00	08:01	14:01	07:01	08:02
Br7	01:01/04N	29:02	08:01	44:03	07:01	16:01
Br8	02:01	29:02:00	44:02:00	44:03:00	05:01/03	16:01
Br10	02:01	23:01	40:02:00	44:03:00	02:02	04:01
Br14	01:01/04N	02:01	07:02	15:01	03:04	07:02
Br15	01:01/04N	29:02	08:01	44:03	07:01	16:01
Br16	01:01/04N	02:01	08:01	44:02	05:01/03	07:01
Br17	02:01	02:01	15:01	51:01	03:03	15:02
Br18	24:02:00	24:02:00	14:02	35:03	04:01	08:02
Br19	02:01	26:01	38:01	44:02	05:01/03	12:03
Br20	01:01/04N	33:05	14:02	57:01	06:02	08:02
Br21	02:01	31:01	15:01	40:01	03:04	03:04
Br22	02:01	02:01	15:01	51:01	02:02	03:04
Br23	01:01/04N	02:01	08:01	15:01	03:03	07:01
Br26	02:01	03:01	07:02	14:01	07:02	08:02
Br27	01:01/04N	24:02	08:01	52:01	07:01	12:02
Br29	02:01	68:01	08:01	51:01	06:02	07:01
Br30	01:01/04N	02:01	40:01	57:01	03:04	06:02
Br31	02:01	02:01	37:01	44:02	05:01/03	06:02
Br32	01:01/04N	02:01	08:01	44:02	05:01/03	07:01
Br37	01:01/04N	02:01	08:01	44:02:00	07:01	12:03
Br24	02:01	02:01	08:01	44:02:00	05:01/03	07:01
Br25	02:01	02:05	07:02	49:01:00	07:01	07:02

Table S4: CDR3α and CDR3β amino acid sequences of the TCRs from the T cell clones isolated from patient Br1 and Br23. Related to STAR Methods and Figure 2.

T cell clone	CDR3α	CDR3β
Br1 clone 1	CLVGEDKLVF	CATHQGAGEQYF
Br1 clone 2	CAASGSNTGNQFYF	CASSSQAGIQTDTQYF
Br23 clone 3	CLVGSNDYKLSF (97%)<br>CAVPRKGRPSNTGKLIF (3%)	CASSQVARDTDTYYGYTF
Br23 clone 4	CALDSNYQLIW	CSGPSLPGGYTF

Table S5: CDR3 amino acid sequences of the TCR alpha and beta chains that are shared between tumor-reactive T cell lines. Related to Figure 3.

Patients	TCR chain	CDR3 sequence
Br6, Br8	alpha	CAALNYGGSQGNLIF
Br8, Br37	alpha	CAASSGGYQKVTF
Br7, Br8	alpha	CALNDYKLSF
Br26, Br27	alpha	CAMREGQMGDDKIIF
Br8, Br23	alpha	CAMREGYNDMRF
Br7, Br8	alpha	CAVNTGGFKTIF
Br7, Br8	alpha	CAVNTNAGKSTF
Br16, Br37	alpha	CAVPNDYKLSF
Br6, Br10	alpha	CAVRGDYKLSF
Br6, Br8	alpha	CVVSEGSGTYKYIF
Br6, Br8	beta	CASSLAGGAYEQYF
Br8,Br10	beta	CASSLGGSSTDTQYF
Br6, Br37	beta	CASSLGRNTEAFF
Br8, Br37	beta	CASSRFTDTQYF

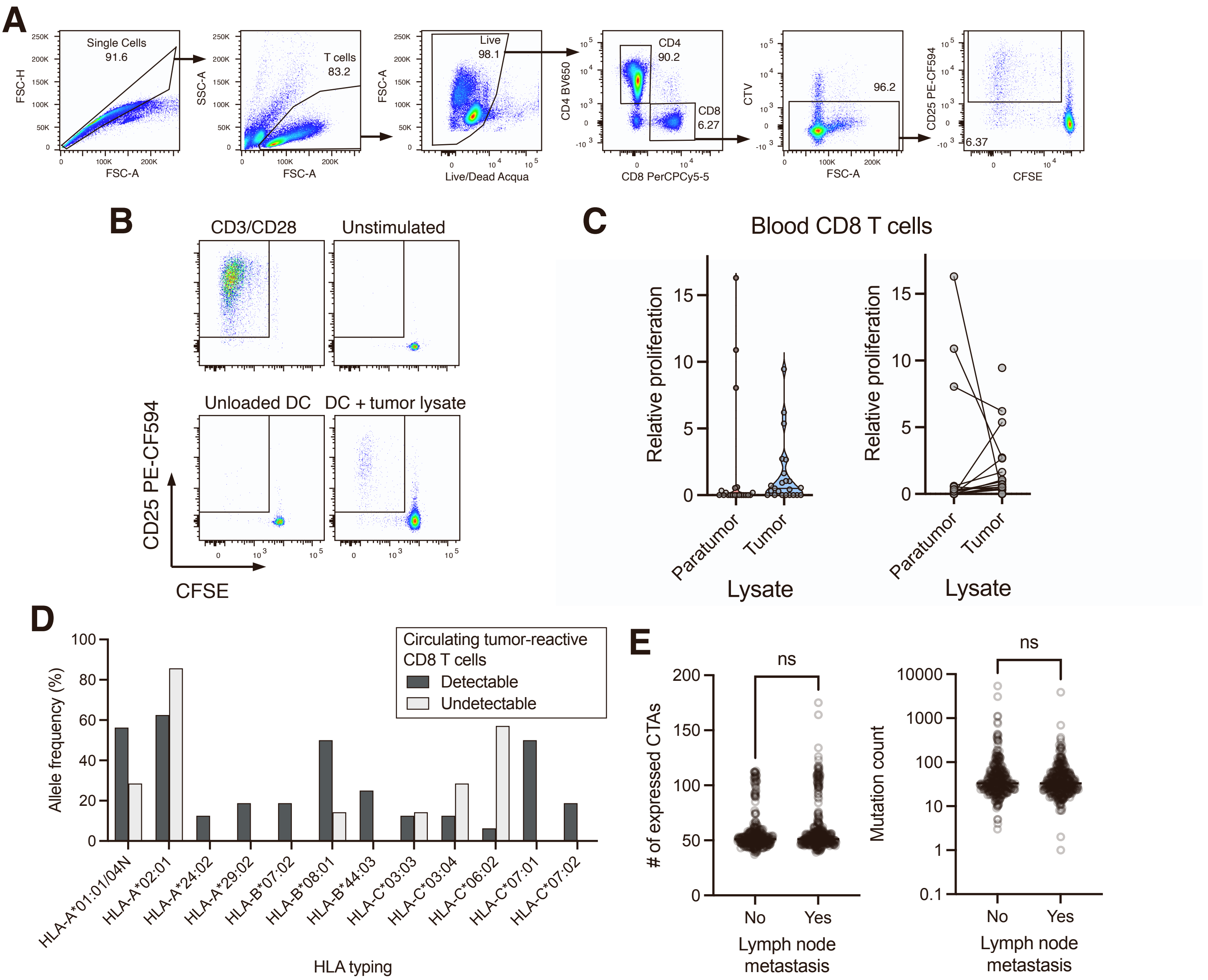

Figure S1: Detection of blood tumor-reactive CD8 T cell responses. (A) Gating strategy of the T cell proliferation assay. (B) Representative dot plots of CFSE and CD25 expression after proliferation assay. (C) Graph showing the normalized percentage of CFSElowCD25+ CD8 T cells after culture with DC loaded with tumor lysate (n = 23) or paratumor lysate (n = 21). (D) Graph showing HLA class I allele frequency, which corresponds to the percentage of patients within either the detectable (n = 16; dark bars) or undetectable (n = 7; light bars) group that have a specific HLA allele. (E) Number of expressed CTAs (p=0.0629) and mutation count (p=0.1726) in TCGA patients with (n=383) and without (n=334) lymph node metastasis. Mann-Whitney test. Related to Figure 1.

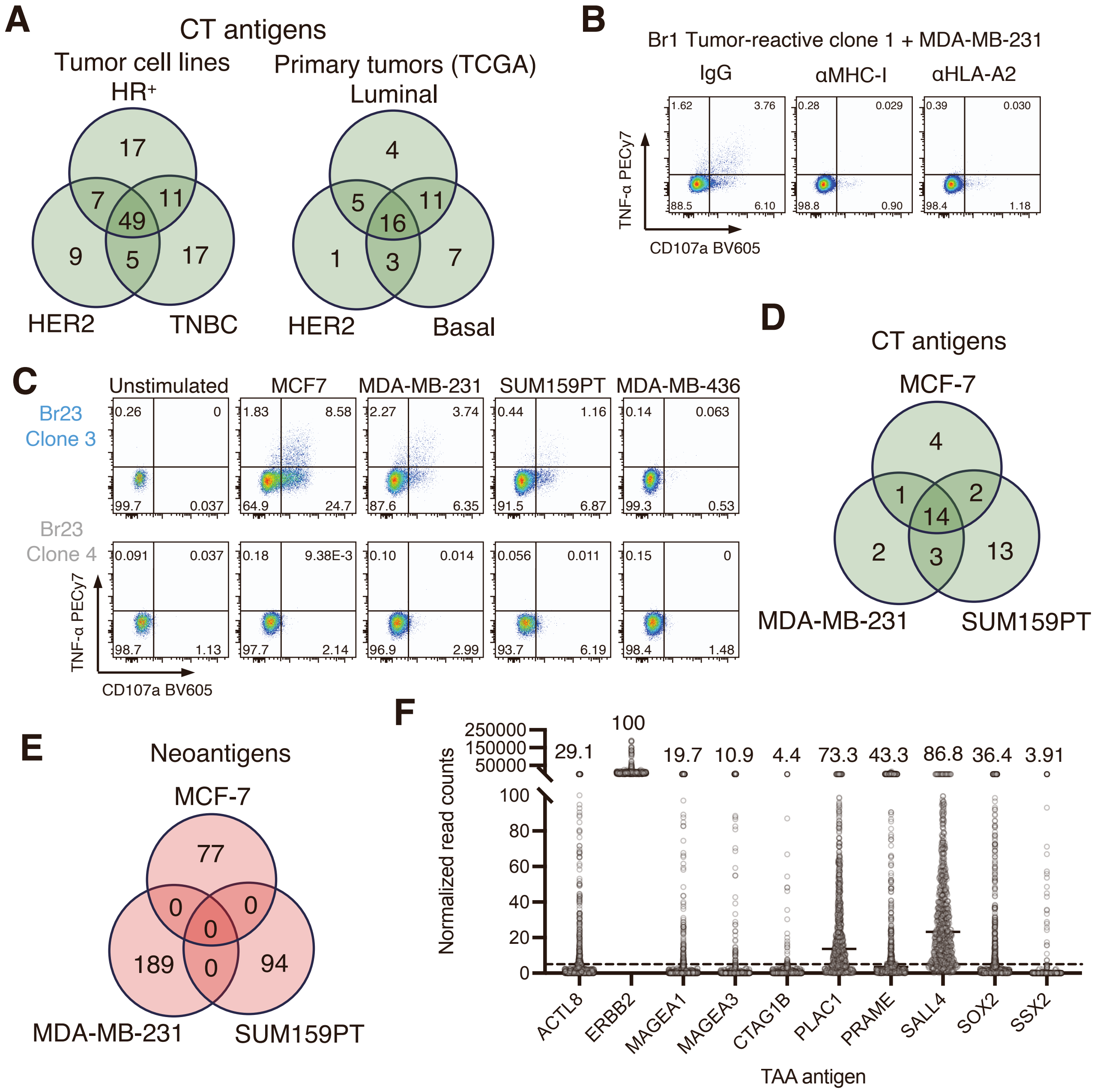

Figure S2: Expression of tumor antigens and specificity of tumor-reactive CD8 T cell clones. (A) Venn diagram showing the number of CTA expressed and shared between cell lines (left) or primary tumors (right) of different subtypes, using the TRON and TCGA dataset, respectively. Number of primary tumors analysed: Luminal n=696; HER2 n=78; Basal n=171. Number of cell lines analysed: HR+ n=9; HER2 n=9; TNBC n=17 (B) Dot plots showing CD107a and TNF-α expression on Br1 tumor-reactive T cell clone 1 cocultured with MDA-MB-231 in the presence of antibodies to block MHC-I, HLA-A2 or an isotype control antibody. (C) Representative graph showing CD107a and TNF- expression on Br23 tumor-reactive T cell clones cultured in the presence of different breast cancer cell lines. (D) Venn diagram showing the number of CTA expressed and shared by each of three breast cancer cell lines (MCF-7, MDA-MB-231, SUM159PT), as determined using the TRON cell line portal dataset. (E) Venn diagram showing the number neoantigens expressed and shared by each of the three breast cancer cell lines, as determined using the TRON cell line portal dataset. (F) Normalized RNA expression of 10 tumor-associated antigens (TAA) in breast cancer patients of Luminal A, Luminal B and Normal molecular intrinsic tumor subtypes from the TCGA dataset (n=818). Related to Figure 2.

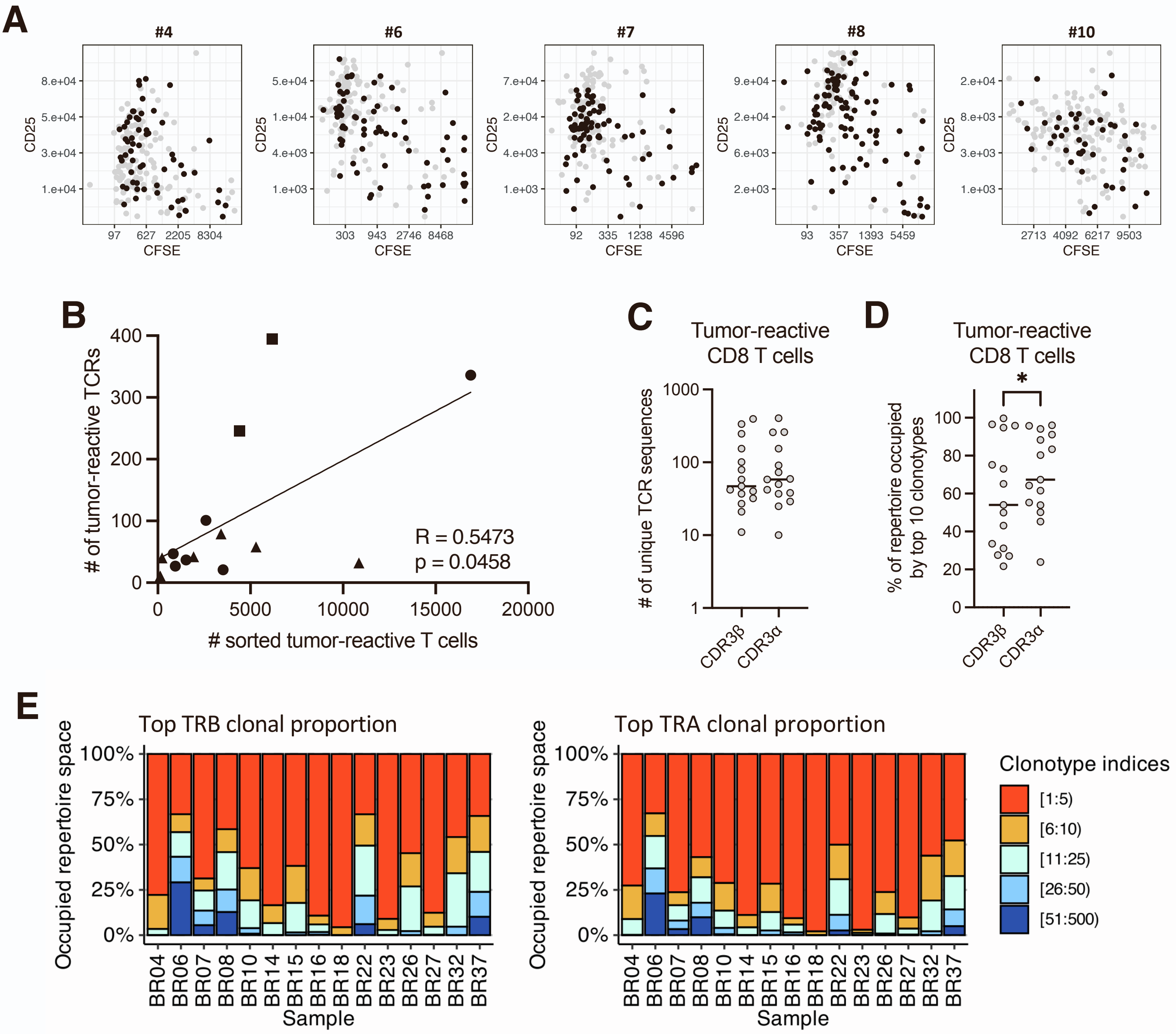

Figure S3: Analysis of tumor-reactive CD8 TCRs. (A) Dot plot showing the CFSE dilution and CD25 expression of each sorted individual CD8 T cell that was proliferating in the presence of the tumor lysate. Each single cell was expanded with feeders, and those that were successfully expanded into T cell clones are marked in black. (B) Correlation analysis between the number of tumor-reactive CDR3β and the number of sorted tumor-reactive T cells. Correlation analysis was performed using non-parametric Spearman rank correlation (p=0.0458). The shape of the dots represents the breast cancer subtype (circle = ductal; triangle = lobular; square = mucinous). (C) Number of unique CDR3 alpha and beta sequences in the tumor-reactive blood CD8 T cell lines. Each dot represents a different patient (n = 15; p=0.6001; Wilcoxon matched-pairs signed rank test). (D) Percentage of the repertoire occupied by the top 10 CDR3 alpha or beta clonotypes on the tumor-reactive T cell lines (n = 15; p=0.0147; two-tailed paired t-test). (E) Horizontal bar graph showing the proportion of the repertoire space occupied by the 5 most abundant clonotypes (red), or by the clonotypes with the indicated indices, in the TRB (left) or TRA (right). Related to and Figure 3.

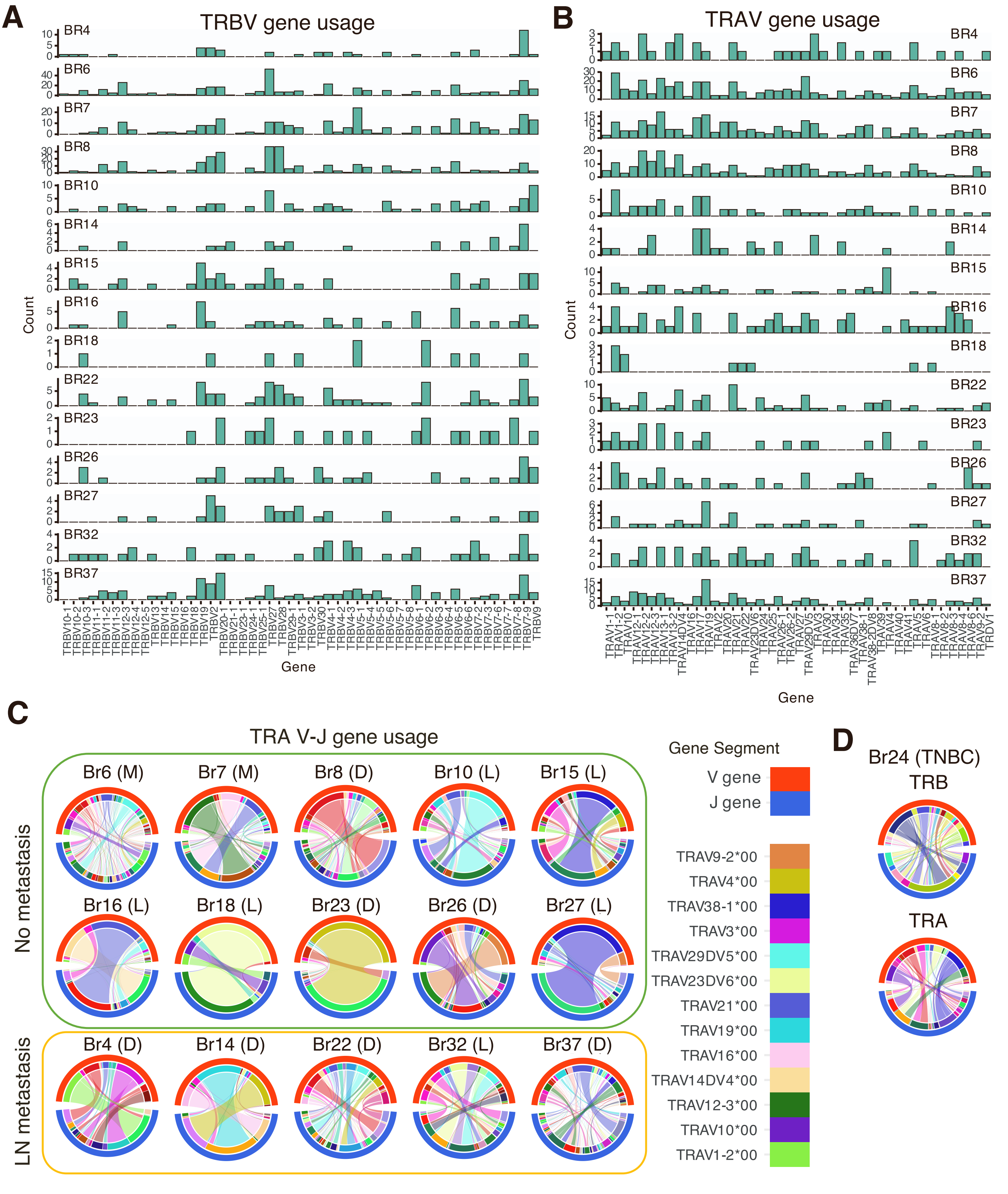

Figure S4: Tumor-reactive CD8 T cell repertoire diversity. (A) Plot showing the number of tumor-reactive clonotypes bearing each TRBV gene fragment in each patient. (B) Plot showing the number of tumor-reactive clonotypes bearing each TRAV gene fragment in each patient. (C)) V-J rearrangement circos plots of the T cell receptor alpha chain (TRA) in tumor-reactive T cell lines derived from each patient. Each plot represents the distribution of V (red outer arc) and J (blue outer arc) gene segment usage, with the connecting lines indicating specific V-J gene rearrangements for each TCR clonotype. The width of connecting lines reflects the relative abundance of that specific clonotype, with the color corresponding to the used TRAV segment. In parentheses next to each patient ID is the subtype of the tumor (L = lobular, D = ductal, M = mucinous). Samples are grouped based on the presence or absence of lymph node metastasis. (D) V-J rearrangement circos plot of the T cell receptor beta (TRB) alpha chain (TRA) of the tumor-reactive T cell line from a triple-negative breast cancer (TNBC) patient (Br24). Related to Figure 3.

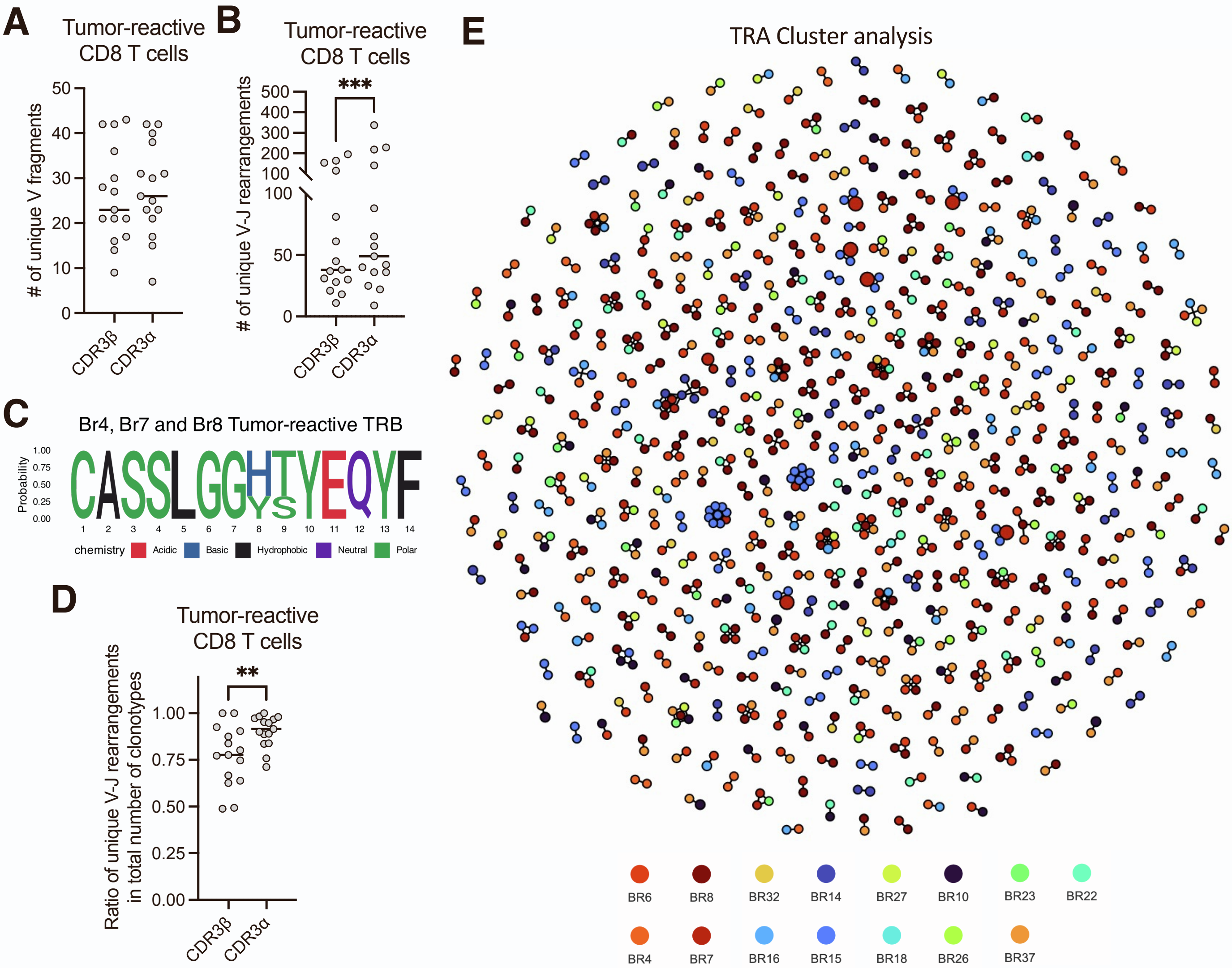

Figure S5: Diversity of TCR alpha and beta chain. (A) Number of unique V gene fragments used by the alpha and beta chain of tumor-reactive TCRs (n = 15; p=0.0777; two-tailed paired t-test). (B) Number of unique V-J rearrangements used by the alpha and beta chain of tumor-reactive TCRs (n = 15; p=0.0001; Wilcoxon matched-pairs signed rank test). (C) Sequence logo plot of a tumor-reactive CDR3β that is similar between three patients. (D) Ratio of unique V-J rearrangements used by the alpha and beta chain of tumor-reactive TCRs, in the total number of clonotypes (n = 15; p=0.0056; two-tailed paired t-test). (E) TRA cluster analysis of tumor-reactive CD8 T cells using the GLIPH2 algorithm. Each color represents a different patient. The link highlights TCRs that are similar based on global alignment of the CDR3α. Related to Figure 3.

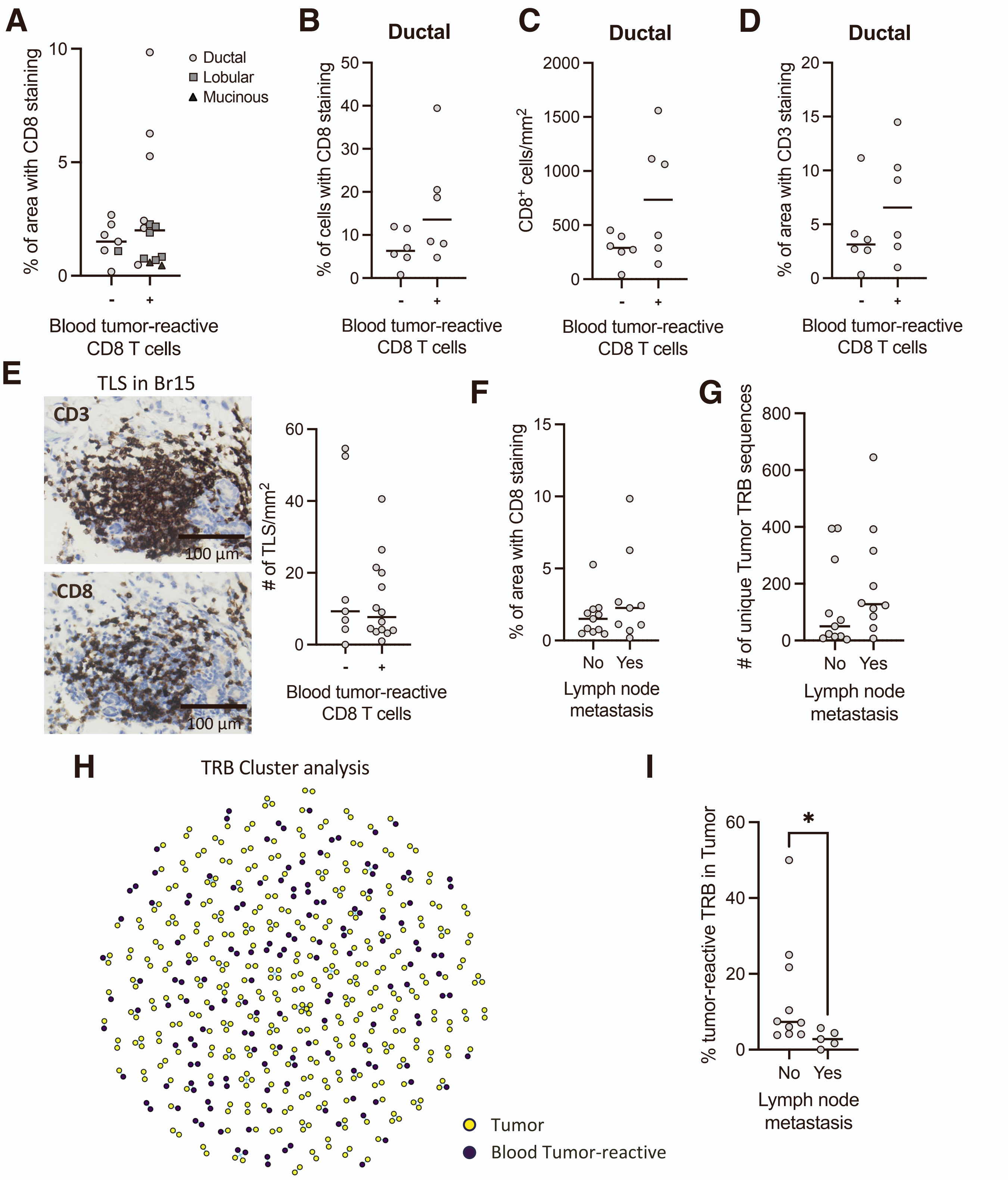

Figure S6: Analysis of tumor-reactive TILs. (A) Percentage of the total tumor area with CD8 staining in patients with (n=14) and without (n=7) a detected blood tumor-reactive T cell response (p=0.7433; Mann-Whitney test). (B) Percentage of $CD8^+$ cells in 10 randomly selected tumor areas in ductal breast cancer patients with (n=6) and without (n=6) a detected blood tumor-reactive T cell response (p=0.1085; two-tailed unpaired t-test). (C) Number of $CD8^+$ cells per mm^3 of tumor area, calculated using 10 randomly selected areas inside ductal breast tumors, in ductal patients with (n=6) and without (n=6) a detected blood tumor-reactive T cell response (p=0.0732; two-tailed unpaired t-test). (D) Graph showing the lack of difference in the percentage of the total tumor area with CD3 staining in ductal patients with (n=6) and without (n=6) a detected blood tumor-reactive T cell response (p=0.2904; two-tailed unpaired t-test). (E) Representative IHC of the CD3 and CD8 staining in a region characteristic of tertiary lymphoid structures (TLS), and graph showing the number of TLS present in patients with (n=14) and without (n=7) a detected blood tumor-reactive T cell response (p=0.5846; Mann-Whitney test). (F) Percentage of the total tumor area with CD8 staining in patients with (n=9) and without (n=11) lymph node metastasis (p=0.2947; Mann-Whitney test). (G) Number of unique TRB sequences in tumor-reactive blood-derived CD8 T cell lines in patients with (n=10) and without (n=11) lymph node metastasis (p=0.1971; Mann-Whitney test). (H) TRB cluster analysis of blood tumor-reactive CD8 T cells (dark blue) and TILs (yellow) using the GLIPH2 algorithm. The link highlights TCRs that are similar based on global alignment of the CDR3β. (I) Graph showing the percentage of TRB sequences that are similar, based on the GLIPH2 analysis, or identical to tumor-reactive TRBs, in patients with (n=5) and without (n=10) lymph node metastasis (p=0.0193; Mann-Whitney test). Related to Figure 4.

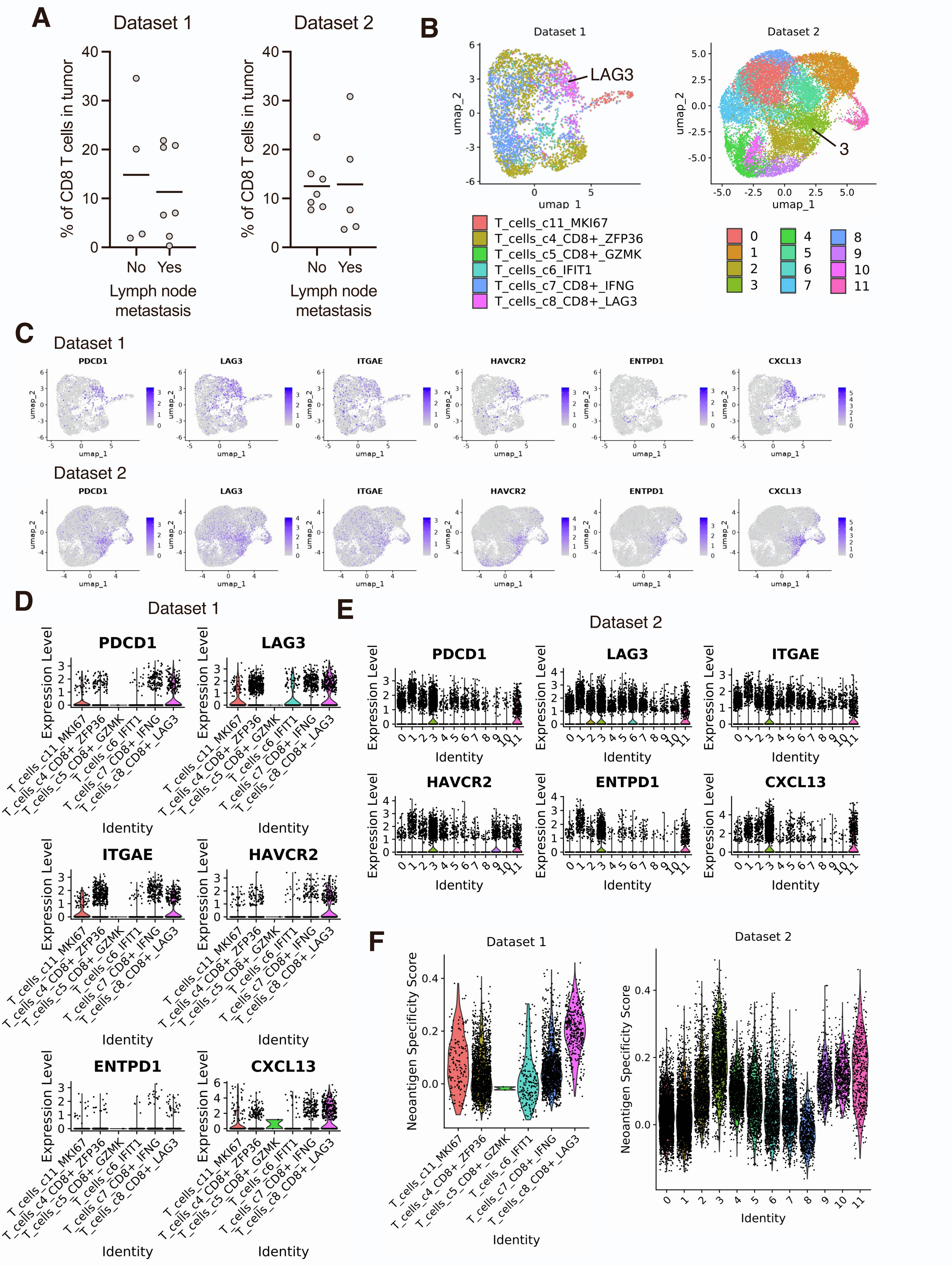

A
Dataset 1
Dataset 2
% of CD8 T cells in tumor
No Yes
Lymph node metastasis
B
Dataset 1
Dataset 2
LAG3
3
umap_1
umap_2
T_cells_c11_MKI67
T_cells_c4_CD8+_ZFP36
T_cells_c5_CD8+_GZMK
T_cells_c6_IFIT1
T_cells_c7_CD8+_IFNG
T_cells_c8_CD8+_LAG3
C
Dataset 1
PDCD1 LAG3 ITGAE HAVCR2 ENTPD1 CXCL13
Dataset 2
D
Dataset 1
Expression Level
Identity
E
Dataset 2
F
Dataset 1
Dataset 2
Neoantigen Specificity Score
Identity

Figure S7: Analysis of two independent scRNA-seq datasets. (A) Graph showing the lack of difference in the percentage of CD8 T cells in the tumor of patients with (Dataset 1: n=7; Dataset 2: n=5) and without (Dataset 1: n=4; Dataset 2: n=7) a detected blood tumor-reactive T cell response (Dataset 1: p=0.6482; Dataset 2: p=0.9391; two-tailed unpaired t-test). (B) UMAP plot of scRNA-seq from the two datasets colored by cluster ID. (C) UMAP plots of the two datasets colored by RNA expression of six different genes that correlates with neoantigen specificity. (D) Violin plots of dataset 1 showing the expression of six genes divided by cluster. (E) Violin plots of dataset 2 showing the expression of six genes divided by cluster. (F) Violin plots of dataset 1 and 2 showing the neoantigen specificity score divided by cluster. Related to Figure 5.
