# Supplementary material for: Tumor-specific CD8 T cell characterization in HR+ breast cancer reveals an impaired antitumoral response in patients with lymph node metastasis
Source: Cell Rep Med. 2025 Jul 28;6(8):102252. doi: 10.1016/j.xcrm.2025.102252 (PMC12432377; doi:10.1016/j.xcrm.2025.102252)
Supplement: Document S2. Article plus supplemental information [file mmc2.pdf]

Cell Reports
Medicine

Article

Tumor-specific CD8 T cell characterization in HR$^+$ breast cancer reveals an impaired antitumoral response in patients with lymph node metastasis

Graphical abstract

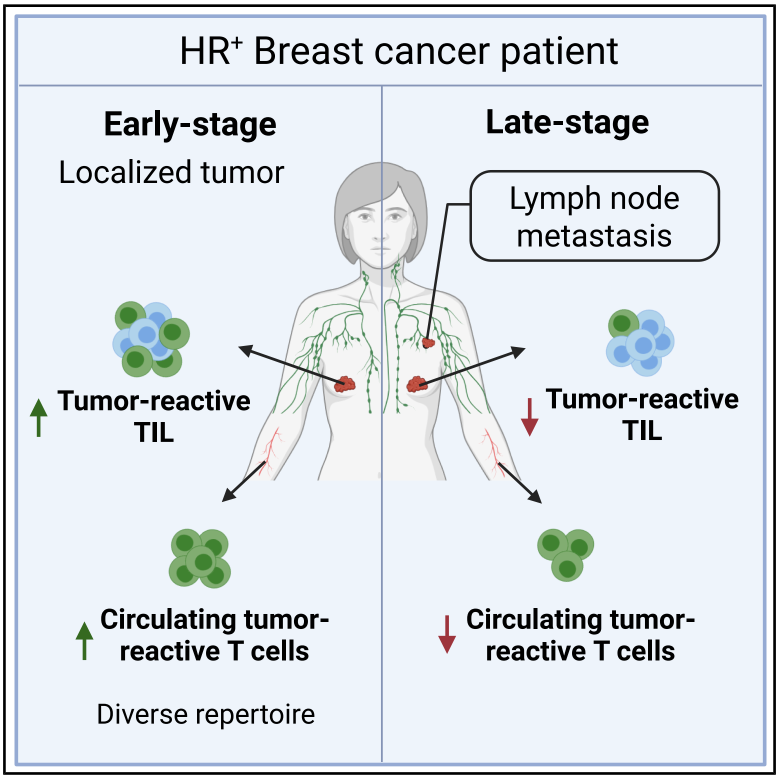

Highlights

- Antitumor CD8 T cells are detected in patients with early-stage breast cancer
- When present, the tumor-reactive blood CD8 T cell repertoire is highly diverse
- Presence of circulating tumor-reactive T cells correlates with CD8 TIL abundance
- Patients with lymph node metastasis have lower frequency of tumor-reactive TILs

Authors

Mariana Pereira Pinho, Elie Antoun, Balraj Sandhar, ..., Yanchun Peng, Asha Adwani, Tao Dong

Correspondence

tao.dong@ndm.ox.ac.uk

In brief

Pinho et al. show that HR$^+$ breast cancer patients with lymph node metastasis have an impaired antitumor CD8 immune response, with less frequency of tumor-reactive TILs. Yet, early-stage patients exhibit a diverse tumor-reactive CD8 T cell repertoire that could be harnessed to develop immunotherapeutic interventions for late-stage HR$^+$ patients.

Pinho et al., 2025, Cell Reports Medicine *6*, 102252
August 19, 2025 © 2025 The Authors. Published by Elsevier Inc.
https://doi.org/10.1016/j.xcrm.2025.102252

CellPress

Cell Reports Medicine

CellPress OPEN ACCESS

Article

Tumor-specific CD8 T cell characterization in HR^+ breast cancer reveals an impaired antitumoral response in patients with lymph node metastasis

Mariana Pereira Pinho,[1,2] Elie Antoun,[2,3] Balraj Sandhar,[1,2] Ting Shu,[2] Fei Gao,[1,2] Xiaobao Yang,[2] Adam Bates,[1,2] Lucia Cerundolo,[4] Megat H.B.A. Hamid,[2] David Maldonado-Perez,[4] Renuka Teague,[4] Eve Warner,[4] Lucinda Winter,[5] Nasullah Khalid Alham,[6,7] Clare Verrill,[4,7] Simon R. Lord,[8] Timothy Rostron,[9] Sally-Ann Clark,[10] Craig Waugh,[10] Paul Sopp,[10] Chris Conlon,[2] Ricardo A. Fernandes,[2] Adrian L. Harris,[8] Yanchun Peng,[1,2,12] Asha Adwani,[11,12] and Tao Dong[1,2,12,13,*]

[1]Medical Research Council Translational Immune Discovery Unit (MRC TIDU), Weatherall Institute of Molecular Medicine (WIMM), University of Oxford, Oxford, UK

[2]Chinese Academy of Medical Sciences (CAMS) Oxford Institute (COI), University of Oxford, Oxford, UK

[3]Centre for Human Genetics, Nuffield Department of Medicine, University of Oxford, Oxford, UK

[4]Nuffield Department of Surgical Sciences, University of Oxford, John Radcliffe Hospital, Oxford, UK

[5]Department of Cellular Pathology, Oxford University NHS Foundation Trust, Oxford, UK

[6]Institute of Biomedical Engineering, Department of Engineering Science, University of Oxford, Oxford, UK

[7]Oxford National Institute of Health Research (NIHR) Biomedical Research Centre, John Radcliffe Hospital, Oxford, UK

[8]Department of Oncology, University of Oxford, Oxford, UK

[9]Sequencing Facility, MRC Weatherall Institute of Molecular Medicine, University of Oxford, Oxford, UK

[10]Flow Cytometry Facility, MRC Weatherall Institute of Molecular Medicine, Radcliffe Department of Medicine, University of Oxford, Oxford, UK

[11]Department of Breast Surgery, Oxford University Hospitals NHS Foundation Trust, Oxford, UK

[12]Senior author

[13]Lead contact

*Correspondence: tao.dong@ndm.ox.ac.uk

https://doi.org/10.1016/j.xcrm.2025.102252

SUMMARY

Most breast cancers express the estrogen receptor (ER), but the immune response of hormone receptor-positive (HR^+) breast cancer remains poorly characterized. Here, dendritic cells loaded with tumor lysate are used to identify tumor-reactive CD8 T cells, which are detected in most HR^+ breast cancer patients, especially those with early-stage tumors. When present, the circulating antitumor CD8 response contains cytotoxic T cells with diverse specificity and T cell receptor (TCR) repertoire. Additionally, patients with blood cancer-specific T cells have significantly more CD8 tumor-infiltrating lymphocytes (TILs). Moreover, tumor-reactive TCR sequences are detected in the tumor, but at a significantly lower proportion in patients with lymph node involvement. Our data suggest that HR^+ breast cancer patients with lymph node metastasis lack tumor-specific CD8 T cells with capacity to infiltrate the tumor at significant levels. However, early-stage patients have a diverse antitumor CD8 response that could be harnessed to develop immunotherapeutic approaches for late-stage HR^+ patients.

INTRODUCTION

Breast cancer is the second most common cancer worldwide, and it is the leading cause of cancer-related death in women.[1] The expression of the estrogen receptor (ER), progesterone receptor, and human epidermal growth factor receptor 2 (HER2) on breast tumors is used to subdivide patients and guide treatment. Immune checkpoint inhibitors (ICIs) are the standard of care for tumors that lack expression of all three molecules (triple-negative), but it is not used to treat the more common hormone receptor-positive (HR^+) cancers.[2] The limited breast cancer response to ICIs in comparison to other types of cancer such as melanoma and renal cell carcinoma has driven the current surge in studies aiming to analyze the immune response and elucidate the mechanisms of response and resistance in those patients.

T cells are a key component of the antitumor immune response, with CD8 T cells capable of directly killing cancer cells by recognizing tumor antigens.[3] Historically, breast cancer has been considered as an immunologically "cold" tumor, due to its low tumor T cell infiltration.[4] Additionally, breast cancers are known to have a low expression of tumor-associated antigens,[5] as well as have a low tumor-mutation rate, therefore restricting the quantity of possible neoantigen epitopes.[6,7] However, recent

 © 2025 The Authors. Published by Elsevier Inc.
This is an open access article under the CC BY license (http://creativecommons.org/licenses/by/4.0/).

studies have challenged the historical view that breast tumors are immunologically quiescent. To determine the immune landscape of tumors, Thorsson et al. identified six immune subtypes across different cancers and determined the most abundant subtype in breast tumors to be interferon (IFN)-γ dominant. They found that less than 10% of breast tumors associated with the lymphocyte-depleted subtype, and none was assigned the immunologically quiet subtype.[8] Despite the low expression of tumor antigens on breast tumors, we and others have shown that tumor-specific T cells, including those specific for neoantigens, have been consistently found in breast cancer patients, demonstrating that breast tumors are immunogenic.[9–11]

While the existence of tumor-specific T cells in breast cancer patients has been proven, these T cells are still very poorly characterized, especially in HR$^+$ breast tumors. HR$^+$ breast cancer is the most prevalent breast cancer molecular subtype, accounting for approximately 70% of clinical cases.[12] They are characterized by their ER expression and responsiveness to hormonal therapy, but this subtype has the lowest response rate for immunotherapy.[13] The lack of studies investigating the tumor-specific T cell responses naturally generated by HR$^+$ breast tumors has hindered our ability to truly understand the tumor immune interplay in these patients, which could allow for the design of better tailored immunotherapeutic interventions. Nevertheless, studying these cells comes with some specific challenges. Unlike other tumors, T cell responses to only a few tumor antigens expressed in a small proportion of patients have been identified in breast cancer patients.[14–17] Also, the low number of T cells infiltrating the tumor restricts the direct study of these cells.

To overcome the aforementioned challenges, we used tumor lysate as the antigen source to detect circulating T cells specific for any antigen present in the autologous tumor. Using this approach, we successfully characterized the tumor-reactive CD8 T cell receptor (TCR) repertoire in HR$^+$ breast cancer patients. Notably, we detected CD8 T cells with killing capacity and with specificity to known tumor antigen. We further established a correlation between the circulating tumor-reactive response and CD8 T cell tumor infiltration in HR$^+$ breast tumors and showed that circulating tumor-reactive TCRs could be detected in the tumor-infiltrating T cells (TILs). Moreover, our data provide solid evidence that the presence of an effective antitumoral response of HR$^+$ breast cancer tissue depends on the tumor stage. Most patients with lymph node metastasis either did not have a detectable circulating antitumor CD8 T cell response or had a significantly lower proportion of tumor-reactive TIL. However, early-stage patients had a diverse antitumor CD8 response capable of infiltrating the tumor. These data suggest a possible explanation for the low efficacy of checkpoint blockage treatment in metastatic HR$^+$ breast cancer patients and offer insights into the types of immunotherapeutic interventions that might be more effective on these patients.

RESULTS

A circulating tumor-reactive CD8 T cell response can be found in most early-stage HR$^+$ breast cancer patients

In this study, we sought to investigate the tumor-reactive CD8 T cell response in a cohort of HR$^+$ breast cancer patients (n = 23; Table 1). To better characterize the overall tumor-reactive CD8 T cell response in this cohort, a broad antigen-agnostic autologous approach was chosen to identify T cells specific for the patients' own tumor. In brief, autologous monocyte-derived dendritic cells (moDCs) loaded with autologous tissue lysates were used to stimulate circulating T cells (Figures 1A, S1A, and S1B). While just a few patients exhibited proliferation against a lysate generated with paratumor tissue, proliferation against the tumor lysate was observed in 16 out of 23 (69.6%) HR$^+$ breast cancer patients (Figure S1C). Our data show that most HR$^+$ breast cancer patients have a detectable circulating tumor-reactive CD8 T cell response.

Since a proportion of patients did not have a detectable circulating CD8 T cell response, we next evaluated which patients' characteristics correlated with the presence of the antitumor response. All patients with certain major histocompatibility complex (MHC) class I alleles had a detectable antitumor T cell response (e.g., HLA-C*07:01), suggesting that some of the tumor-reactive CD8 T cells might be restricted to these alleles (Figure S1D).

HR$^+$ breast cancer has different histological types, the two major ones being ductal and lobular cancer. While 8 out of 14 (57.1%) patients with ductal tumors had a detectable response, 6 out of 7 (85.7%) lobular tumor-bearing patients showed the presence of circulating tumor-reactive T cells. Additionally, the two patients with mucinous tumors also had a detectable proliferation against the tumor lysate (Figure 1B). Interestingly, a significantly lower response was observed among patients with lymph node metastasis (Figure 1C; p = 0.033). The same was also observed when analyzing patients with ductal histology separately, indicating that the presence of lymph node metastasis correlated with the lack of circulating tumor-reactive CD8 T cells, regardless of the tumors' histological subtype (Figure 1D).

Age, tumor size, and preoperative hormonal treatment did not correlate with the presence or absence of a detectable antitumoral response (Table S1). It is important to note that no patients included in the study had diagnosed distant metastasis at the time of surgery. Patient Br17 was diagnosed with distant metastasis around 1 month after surgery. When analyzing The Cancer Genome Atlas (TCGA) dataset,[18] no difference in the number of expressed cancer testis antigens (CTAs) or mutation count was observed in patients with or without lymph node metastasis (Figure S1E), indicating that the different antitumor response might not be due to differences in antigen load.

Taken together, our results showed that the majority of early-stage HR$^+$ breast cancer patients, especially those with lobular and mucinous tumors, had a detectable tumor-reactive CD8 T cell response in the blood.

Tumor-reactive CD8 T cells can kill tumor cells and contain CTA-specific T cells

In order to confirm the tumor reactivity of the proliferating cells, we used established commercial breast cancer cell lines, as it was not possible to generate autologous tumor cells in sufficient numbers. For this analysis, we selected two patients for which we had a combination of breast cancer cell lines that collectively covered at least 5 of the 6 MHC class I alleles of the patient

Table 1. Characteristics of the HR$^+$ breast cancer patients included in the study

Patient ID	Age	Histological subtype	Lymph node metastasis	Grade	Preoperative treatment
Br8	86	ductal	ND	3	letrozole
Br1	52	ductal	no	3	no
Br20	83	ductal	no	2	letrozole
Br23	72	ductal	no	3	no
Br26	65	ductal	no	3	no
Br29	51	ductal	no	1	no
Br4	72	ductal	yes	3	no
Br14	76	ductal	yes	3	no
Br17	57	ductal	yes	3	no
Br21	65	ductal	yes	2	no
Br22	65	ductal	yes	3	no
Br30	71	ductal	yes	3	no
Br31	54	ductal	yes	2	no
Br37	39	ductal	yes	3	no
Br10	63	lobular	no	2	no
Br15	75	lobular	no	2	no
Br16	55	lobular	no	2	no
Br18	67	lobular	no	2	no
Br27	73	lobular	no	2	no
Br19	70	lobular	yes	2	no
Br32	75	lobular	yes	2	letrozole
Br6	65	mucinous	no	2	no
Br7	71	mucinous	no	2	no

ND, not determined.

(Tables S2 and S3). Although the patients had HR$^+$ breast tumors, we used cell lines from different breast cancer subtypes, as cell lines and primary tumors across different subtypes share expression of CTAs (Figure S2A). Tumor-reactive CD8 T cell clones from these patients were generated by single-cell sorting of the proliferating (CFSE^{low}CD25$^+$) CD8 T cells activated with the tumor lysate, subsequently expanding them *in vitro* with allogeneic feeders. The clonality of the T cell clones was confirmed by TCR sequencing (Table S4).

Two T cell clones generated from an HLA-A*02:01$^+$ patient (Br1) were challenged with breast cancer cell lines with different HLA typing (Table S2), and their response was measured by intracellular cytokine staining (ICS). The first clone reacted to an HLA-A*02:01$^+$ cell line (MDA-MB-231), while the second clone reacted to an HLA-A*02:01$^-$ cell line (HCC1937) (Figure 2A). The cell line HCC1937 has three MHC-I alleles (HLA-A*24:02, HLA-B*07:02, and HLA-C*07:02) that match the patient's alleles (Table S3). The HLA-A2 restriction of the first clone was confirmed by using an antibody that directly blocks the T cell recognition of HLA-A2 on the target cells (Figures 2B and S2B). Consistently, these two T cell clones were able to kill the same cancer cell lines to which they showed a response in the ICS assay (Figures 2C and 2D).

Two additional T cell clones from a different patient (Br23) were tested. One T cell clone showed specificity to three HLA-A*02:01$^+$ cell lines (MCF-7, MDA-MB-231, and SUM159PT), while the other did not respond to any of the cell lines tested (Figures 2E, 2F, and S2C). Data analysis using the TRON cell line portal (TCLP)[19] showed that no neoantigen was shared between these three lines, while at least 14 known CTAs were expressed by all three cell lines (Figures S2D and S2E). This shows that some of the tumor-reactive CD8 T cells isolated using the tumor lysate might be specific for antigens that are only present in the autologous tumor and thus do not show reactivity to any tumor cell line. Also, some isolated clones could be specific for antigens that are shared between different breast tumors, thereby having greater potential for clinical applications. To note, the clone that reacted to the three cell lines (Br23 clone 3) expressed a second productive TCR alpha chain, albeit at low levels, that could be contributing to the reactivity pattern of this clone (Table S4).

Attempts were made to determine the antigen specificity of the tumor-reactive CD8 T cells. Unfortunately, breast cancer antigens that induce T cell responses in a high proportion of HR$^+$ breast cancer patients have not been broadly described. Thus, in this study, we selected 10 promising tumor-associated antigens based on public breast tumor cell expression data (Figure S2F). We used overlapping peptides from these antigens against the tumor-reactive T cell lines generated from bulk sorting and expansion of CFSE^{low}CD25$^+$ CD8 T cells from 15 patients. Only 3 tumor-reactive circulating CD8 T cell lines showed responses, which were limited to two CTA (PLAC1 and ACTL8) overlapping peptide pools, reiterating the challenge of finding relevant breast cancer antigens (Figure 2G).

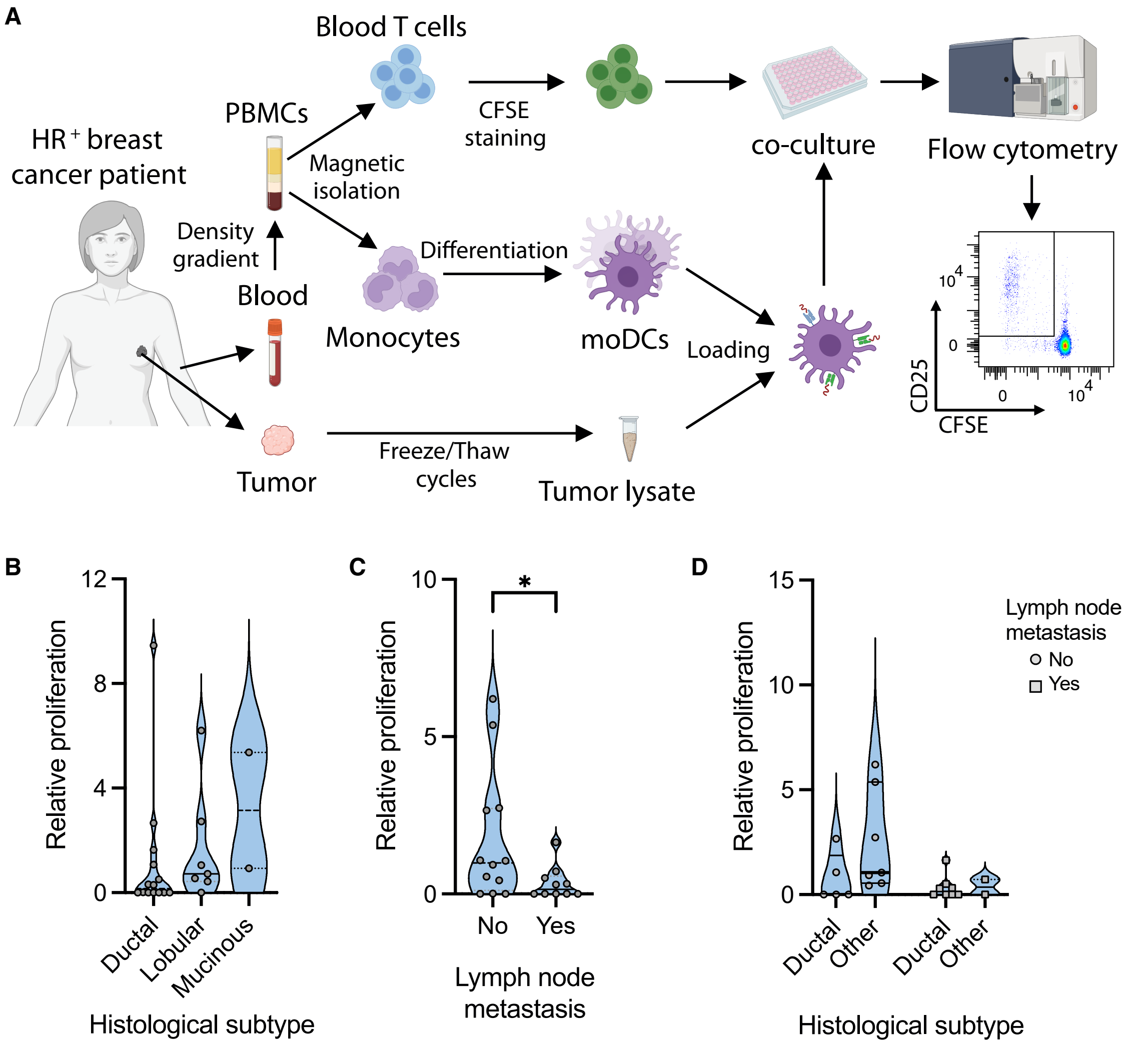

Figure 1. A circulating tumor-reactive CD8 T cell response can be found in most early-stage HR+ breast cancer patients

(A) Schematic of proliferation assay and representative flow cytometry dot plot. moDCs were generated from blood monocytes, loaded with tumor lysates and cocultured with blood T cells.

(B) Graph showing relative T cell proliferation against the tumor lysate, in patients with different histological subtypes (ductal $n = 14$; lobular $n = 7$; mucinous $n = 2$).

(C) Relative proliferation against the tumor lysate in patients divided by the presence of lymph node metastasis (yes $n = 10$; no $n = 12$; $p = 0.033$; Mann-Whitney test).

(D) Graph of the tumor lysate-induced proliferation in patients with different histological subtypes and lymph node metastasis status (ductal no metastasis $n = 5$; ductal with lymph node metastasis $n = 8$; other subtype no metastasis $n = 7$; other subtype with lymph node metastasis $n = 2$). $^{*}p < 0.05$.

These data confirm that tumor-reactive T cells isolated from the peripheral blood of breast cancer patients can kill tumor cells and contain CTA-specific clonotypes.

The circulating tumor-reactive CD8 T cell population consists of a diverse, but mostly private, repertoire

To gain an understanding of the TCR diversity of the tumor-reactive T cell response detected in HR^{+} breast cancer patients, we performed TCR sequencing of the tumor-reactive CD8 T cell lines generated from the blood of 15 patients that had a detectable circulating antitumor T cell response. The tumor-reactive CD8 T cell lines were generated by bulk sorting the proliferating (CFSE^{low}CD25^{+}) CD8 T cells stimulated with tumor lysate-loaded dendritic cell (DC), subsequently expanding them *in vitro* with allogeneic feeders.

Recognizing that *in vitro* expansion can skew TCR diversity, we first evaluated the feasibility of successfully expanding T cells that had exhibited varying levels of proliferation and CD25 upregulation in response to the tumor lysate stimulation (Figure S3A). While we acknowledge that some degree of clonal loss is unavoidable, the ability to expand clones with distinct activation profiles provided confidence that a representative portion of the initial tumor-reactive repertoire was preserved.

CellPress
OPEN ACCESS

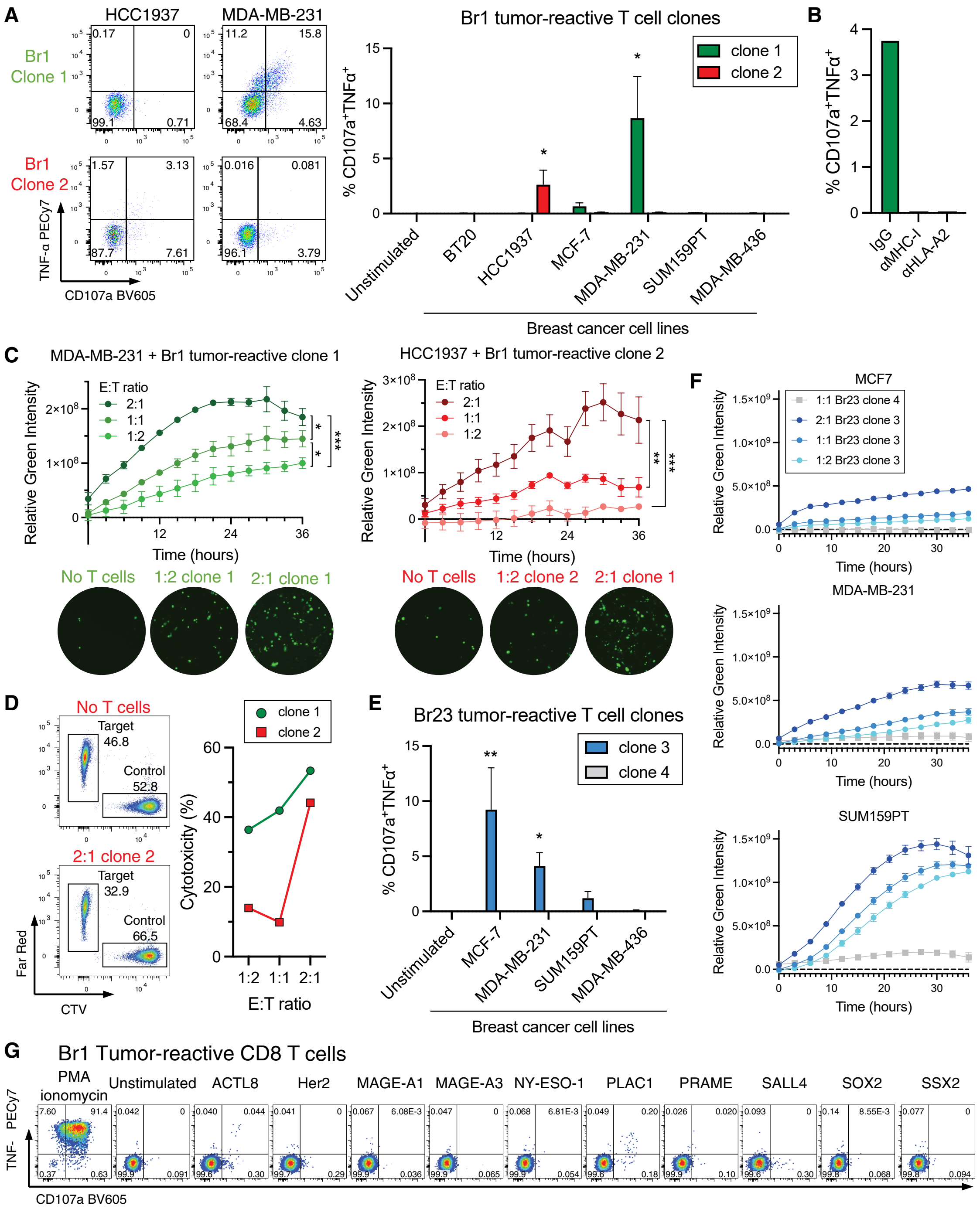

Figure 2. Tumor-reactive CD8 T cells can kill tumor cells and contain CTA-specific T cells

(A) CD107a and TNF-α expression on Br1 tumor-reactive T cell clones cultured in the presence of different breast cancer cell lines in three independent experiments ($n = 3$). Data are represented as the mean ± SEM. Kruskal-Wallis test with Dunn's multiple-comparison tests. Br1 clone 1: MDA-MB-231 vs. unstimulated $p = 0.0277$; Br1 clone 2: HCC1937 vs. unstimulated $p = 0.0269$.

(B) CD107a and TNF-α expression on Br1 tumor-reactive T cell clone 1 cocultured with MDA-MB-231 in the presence of antibodies to block MHC-I, HLA-A2, or an isotype control antibody ($n = 1$).

(C) Killing ability of Br1 tumor-reactive T cell clones by Incucyte at different effector:target (E:T) ratios. One-way ANOVA with Tukey's multiple-comparison tests on the values after 36 h coculture ($n = 3$). MDA-MB-231 + Br1 clone 1: 2:1 vs. 1:1 $p = 0.0268$; 1:1 vs. 1:2 $p = 0.0163$; 2:1 vs. 1:2 $p = 0.0007$. HCC1937 + Br1 clone 2: 2:1 vs. 1:1 $p = 0.0032$; 2:1 vs. 1:2 $p = 0.0008$. Data are represented as the mean ± SD.

(legend continued on next page)

The number of sorted proliferating T cells against the tumor lysate positively correlated with the number of unique TCRs found in the tumor-reactive T cell lines (R = 0.547; p = 0.046; Figure S3B). We next evaluated the complementarity-determining region 3 (CDR3) sequences of each TCR chain. A median of 47 unique CDR3β and 58 CDR3α sequences were identified per patient, with the 10 most expanded CDR3β clonotypes per patient occupying a median of 54.02% of the repertoire space (Figures S3C–S3E).

No difference in the number of unique tumor-reactive TCR beta chain (TRB) sequences and Shannon diversity index was found between patients with different histological subtypes and lymph node metastasis status (Figures 3A and 3B). This indicates that patients with a detectable circulating antitumor CD8 T cell response share a similar degree of tumor-reactive TCR diversity within the tumor-specific compartment, regardless of clinical characteristics.

Evaluation of the different V and J gene usage in both chains highlighted the diversity of the tumor-reactive CD8 T cell repertoire in these patients (Figures 3C and S4A–S4C). A median of 23 differentTCR beta chain variable (TRBV) fragments and 38 different TRB V-J rearrangements were used by the tumor-reactive CD8 T cells (Figures S5A and S5B). No V or J fragment was shown to be preferentially used across multiple patients, except for the usage of TRBV27*00 by the most expanded Br18 and Br27 tumor-reactive clonotypes. The CDR3β sequences for these two clonotypes were considerably different (Br18 CDR3β: CASSPLGPQETQYF; Br27 CDR3β: CASSKGASGNEQFF), indicating that they probably do not recognize the same antigen and thus are not public. In addition, the diversity of tumor-reactive T cell responses in the HR^+ breast cancer patients was similar to that observed in a patient with triple-negative breast cancer (TNBC), which is the subtype of breast cancer that is regarded to be the most immunogenic (Figures 3C, S4C, and S4D).

Next, we investigated if there were any tumor-reactive T cells with the same TCR detectable in multiple patients. A repertoire overlap analysis identified only four shared CDR3β and nine shared CDR3α (Figure 3D; Table S5). A TCR similarity clustering analysis using GLIPH2 (grouping of lymphocyte interactions by paratope hotspots 2) algorithm uncovered just one trio and 31 pairs of similar CDR3β, most of them (20) containing sequences only found in the same patient (Figures 3E and S5C). Interestingly, although the alpha chain had significantly more V-J rearrangement pairs (Figures S5B and S5D), they also contained more similar sequences revealed by the clustering analysis (Figure S5E). The TCR sequencing data confirm that the tumor-reactive T cell repertoire in HR^+ breast cancer patients is very diverse, albeit mostly private.

The presence of circulating tumor-reactive T cells correlates with increased CD8 T cell infiltration in breast HR^+ tumors

HR^+ breast tumors are known to be poorly immune infiltrated, yet some patients do show substantial CD8 T cell infiltration.[20] This was also observed by immunohistochemistry (IHC) of whole tumor sections in our cohort, where only 3 patients out of 21 showed extensive CD8 T cell staining (Figures 4A, 4B, and S6A). Notably, all three patients had a ductal cancer. The number of CD8 T cells was calculated in 20 patients that had sufficient tumor area to perform the selection of at least 5 random regions of interest (ROIs) of 1 mm^2 each inside the tumor (Figures S6B and S6C). Interestingly, all patients with high CD8 tumor T cell frequency and density had a detectable circulating cancer-specific CD8 T cell response (Figures 4B, S6B, and S6C), but this was not observed for total CD3 T cell staining (Figure S6D).

In breast cancer, it has been shown that T cells tend to aggregate at the tumor invasive margin compared to the tumor center.[21] To evaluate the distribution of the CD8 T cells in our cohort, we compared the CD8 T cell count/mm^2 in tumor and in the non-malignant adjacent region. All patients showed greater CD8 T cell density inside the tumor compared to the non-malignant adjacent tissue, but this was significantly greater in patients with a detectable circulating tumor-reactive CD8 response (p = 0.02; Figure 4C).

Previous studies have found that tertiary lymphoid structures (TLSs) can also be identified in breast tumors and that they can correlate with a better prognosis in breast cancer patients.[22] Yet, in our study, we found that TLS number did not correlate with the presence of an antitumor CD8 T cell response in HR^+ breast cancers (Figure S6E).

Taken together, our data indicated that the presence of a circulating tumor-reactive T cell response positively correlates with CD8 T cell tumor infiltration in HR^+ breast cancer patients.

Tumor-reactive T cells can infiltrate the tissue, but patients with lymph node metastasis have fewer tumor-reactive TILs

The presence of a circulating antitumor response does not imply that the tumor-reactive T cells will be able to migrate to the tissue and infiltrate the tumor. Therefore, we next investigated whether we could identify tumor-reactive T cells within breast tumors. Given that T cell specificity is determined by its TCR, we then assessed the detection of tumor-infiltrating CD8 T cells expressing the same TRB or TCR alpha chain (TRA) as the tumor-reactive T cells identified in the blood (Figure 4D). Across all tumors evaluated, a total of 63 tumor-reactive

(D) Killing ability of Br1 tumor-reactive T cell clone 1 (green) against MDA-MB-231 tumor cells or clone 2 (red) against HCC1937 tumor cells by flow cytometry analysis (n = 1).

(E) CD107a and TNF-α expression on Br23 tumor-reactive T cell clone 3 in three independent experiments (n = 3) and clone 4 (n = 1) cultured in the presence of different breast cancer cell lines. Data are represented as the mean ± SEM. Kruskal-Wallis test with Dunn's multiple-comparison tests. Br23 clone 3: MCF-7 vs. unstimulated p = 0.0074; MDA-MB-231 vs. unstimulated p = 0.0316.

(F) Killing ability of Br23 tumor-reactive T cell clone 3 (blue) and 4 (gray) against cell line MCF-7 (top), MDA-MB-231 (middle), and SUM159PT (bottom). Br23 clone 3: n = 3; Br23 clone 4: n = 2. Data are represented as the mean ± SD.

(G) CD107a and TNF-α expression on Br1 tumor-reactive T cell lines cultured in the presence of overlapping peptide pools for 10 different tumor-associated antigens. ***$p < 0.001$, **$p < 0.01$, *$p < 0.05$.

CellPress
OPEN ACCESS

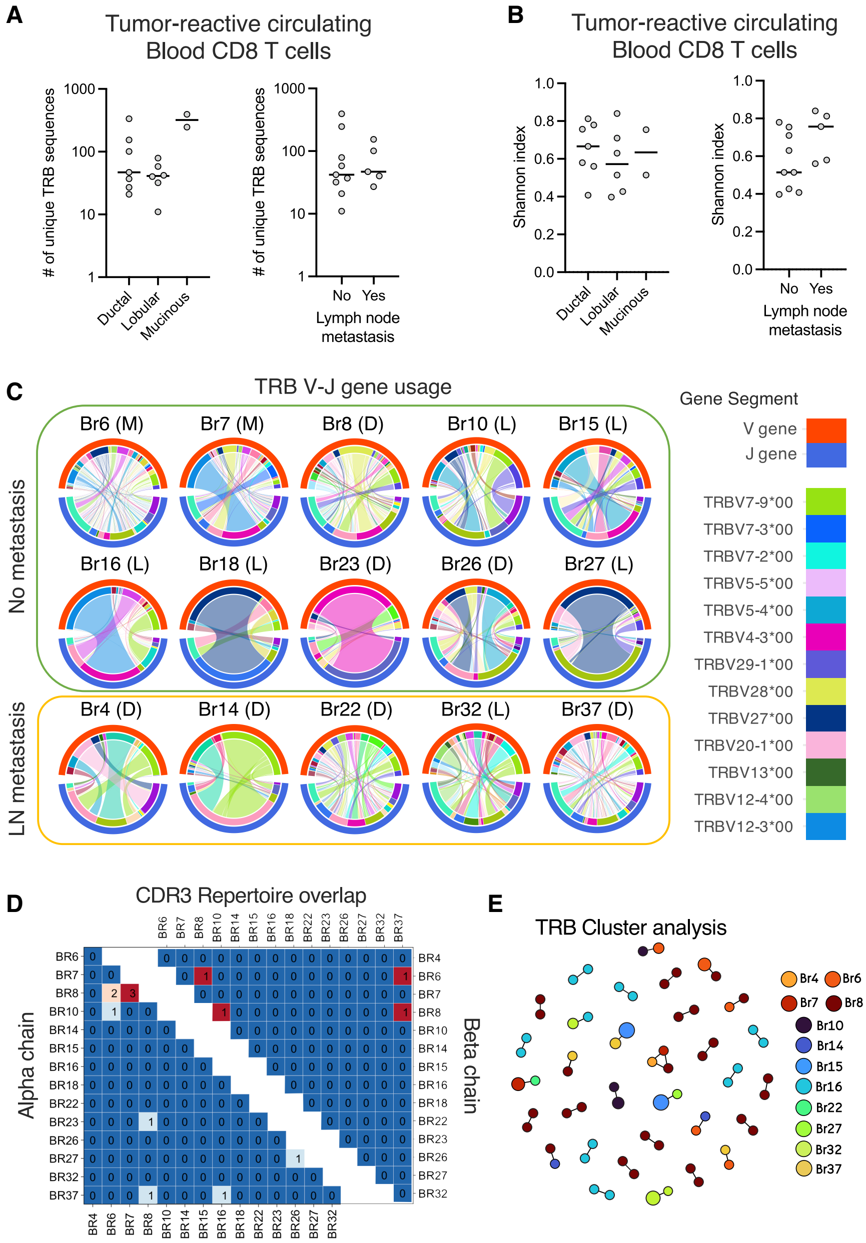

Figure 3. The circulating tumor-reactive CD8 T cell population consists of a diverse, but mostly private, repertoire

(A) Number of unique CDR3 beta sequences in the tumor-reactive blood CD8 T cell lines. Each dot represents a different patient. Patients were divided by histological subtypes (ductal n = 7; lobular n = 6; mucinous n = 2; p = 0.091; Kruskal-Wallis test) or lymph node metastasis (yes n = 9; no n = 5; p = 0.7972; Mann-Whitney test).

(B) Diversity of the tumor-reactive TRBs calculated by the Shannon index and separated by patient's histological subtypes (ductal n = 7; lobular n = 6; mucinous n = 2; p = 0.7062, Kruskal-Wallis test) or lymph node metastasis (yes n = 9; no n = 5; p = 0.0829; Mann-Whitney test).

(C) V-J rearrangement Circos plots of the T cell receptor beta chain (TRB) in tumor-reactive T cell lines derived from each patient. Each plot represents the distribution of V (red outer arc) and J (blue outer arc) gene segment usage, with the connecting lines indicating specific V-J gene rearrangements for each TCR clonotype. The width of connecting lines reflects the relative abundance of that specific clonotype, with the color corresponding to the used TRBV segment. In parentheses next to each patient ID is the subtype of the tumor (L, lobular; D, ductal; M, mucinous). Samples are grouped based on the presence or absence of lymph node metastasis.

(D) Heatmap showing the number of shared CDR3 alpha (left) and beta (right) chains among the tumor-reactive CD8 T cells from different patients.

(E) TRB cluster analysis of tumor-reactive CD8 T cells using the GLIPH2 algorithm. Each color represents a different patient, and the size correlates with the abundance of the clone in the T cell line. The link highlights TCRs that are similar based on global alignment of the CDR3β.

TRB and 73 tumor-reactive TRA were detected in TILs, and the median percentage of TILs with tumor specificity was 3.2% for TRB and 2.8% for TRA. Interestingly, patients without lymph node metastasis had a significantly higher proportion of tumor-reactive TRB and TRA in the tumor, compared to patients with metastasis (p = 0.008; Figures 4E and 4F). The total CD8 infiltration, calculated by both the IHC staining and the number of unique TRB in the tumor, was not associated with lymph node metastasis (Figures S6F and S6G). Additionally, the proportion of pathogen-specific TIL, calculated using curated TRB sequences from the database VDJdb, was not different between patients with or without lymph node metastasis (p = 0.52; Figure 4G).

Lastly, to enable a more robust analysis of the TCR data, a CDR3β clustering analysis using GLIPH2 was performed between circulating tumor-reactive T cells and TILs (Figure S6H). From this analysis, 233 TRB clusters were identified, 119 of them containing tumor-reactive TCRs. This analysis also revealed 45 tumor-derived TRBs, which were similar but not identical to blood-derived tumor-reactive TRBs. When including these similar TRBs to calculate the percentage of tumor-reactive TIL, the median proportion of tumor-reactive TCR in the tumor increased from 3.2% to 5.7%. Yet, patients with lymph node metastasis still exhibit a significantly lower proportion of tumor-reactive TIL (p = 0.019; Figure S6I).

In conclusion, our data show that most patients with lymph node metastasis do not have a detectable circulating CD8 antitumor response, but, even in those who do, the proportion of tumor-reactive TIL is significantly lower compared to patients without lymph node metastasis.

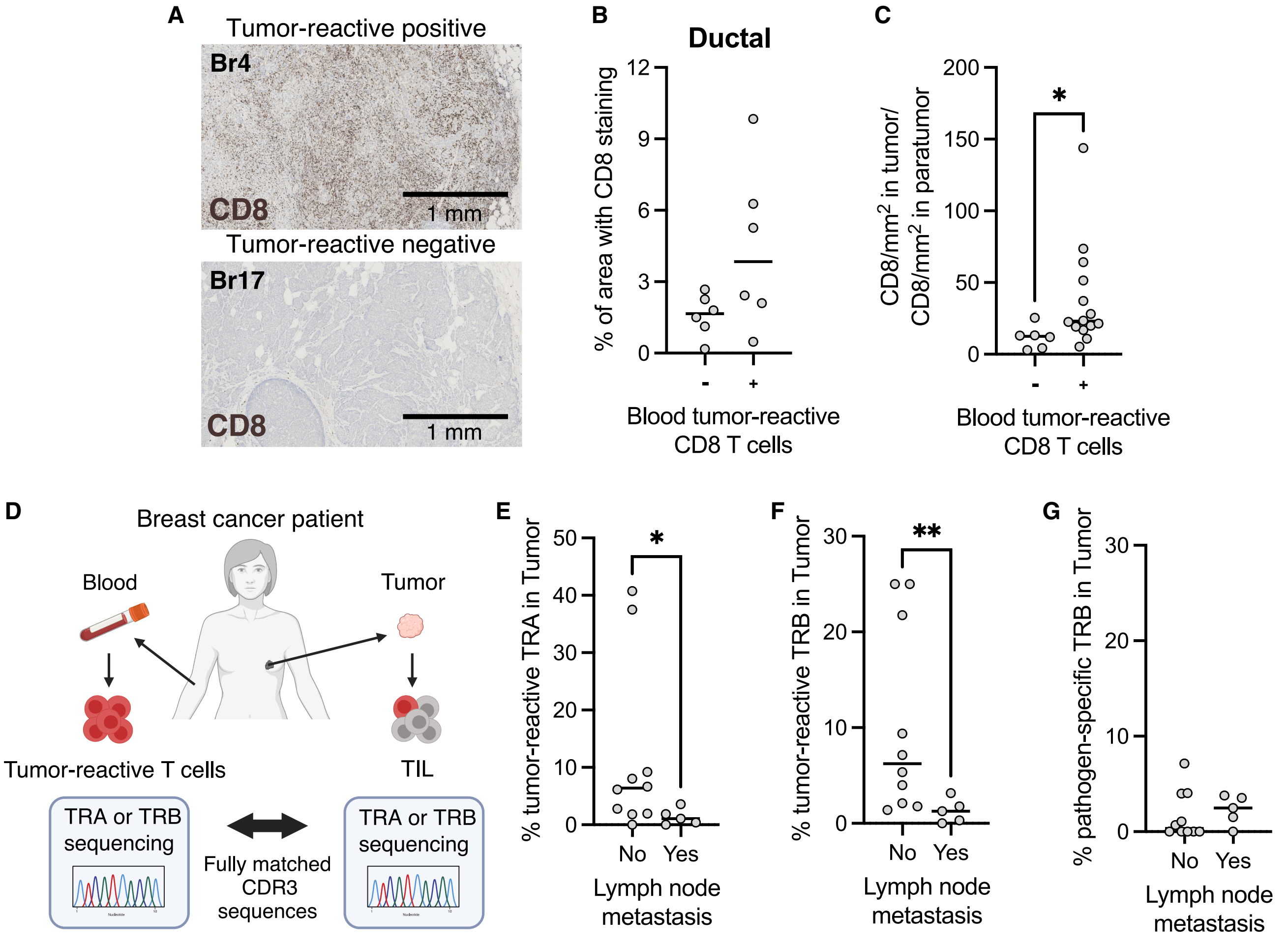

Figure 4. Tumor-reactive T cells can infiltrate the tissue, but patients with lymph node metastasis have fewer tumor-reactive TILs

(A) Representative CD8 immunohistochemistry (IHC) staining in a patient with (tumor-reactive-positive) and without (tumor-reactive-negative) a detected blood tumor-reactive CD8 T cell response.

(B) Percentage of the total tumor area with CD8 staining in ductal breast cancer patients with ($n = 6$) and without ($n = 6$) a detected blood tumor-reactive T cell response ($p = 0.0801$; two-tailed unpaired t test).

(C) Ratio of CD8 T cells present in the tumor compared to the paratumor in breast cancer patients with ($n = 14$) and without ($n = 6$) a detected blood tumor-reactive T cell response ($p = 0.0200$; Mann-Whitney test).

(D) Schematic showing that tumor-reactive TCRs were identified in the tumor by comparing it to blood tumor-reactive TCRs and finding the CDR3 fully matched sequences.

(E) Graph showing the percentage of TRA sequences that fully matched tumor-reactive TRAs, in patients with ($n = 5$) and without ($n = 10$) lymph node metastasis ($p = 0.0416$; Mann-Whitney test).

(F) Graph showing the percentage of TRB sequences that fully matched tumor-reactive TRBs, in patients with ($n = 5$) and without ($n = 10$) lymph node metastasis ($p = 0.0077$; Mann-Whitney test).

(G) Graph showing the percentage of TRB sequences that fully matched pathogen-specific TRBs in the VDJdb, in patients with ($n = 5$) and without ($n = 10$) lymph node metastasis ($p = 0.5201$; Mann-Whitney test). ***$p < 0.001$, **$p < 0.01$, *$p < 0.05$.

CD8 TILs from lymph node-positive HR$^+$ breast cancer patients show reduced neoantigen-specific transcriptional signature in two independent scRNA-seq datasets

To validate the previous finding suggesting a lower frequency of tumor-reactive CD8 TIL in HR$^+$ breast cancer patients with lymph node metastasis, we analyzed two independent single-cell RNA sequencing (scRNA-seq) datasets of breast tumors.[23,24] These datasets were generated by sequencing all cells in the tumor and included detailed lymph node status and a substantial number of HR$^+$ breast cancer patients. First, we selected the HR$^+$ breast cancer patients using the available IHC data, divided the patients by the presence or absence of lymph node metastasis, and filtered to include only CD8 T cells (Figure 5A). Since we have shown that the tumor-reactive TCRs were private to each patient, we used the transcriptional profile of the cells, rather than their TCR sequence, to estimate tumor specificity. Studies have shown that neoantigen-specific CD8 TILs show

CellPress
OPEN ACCESS

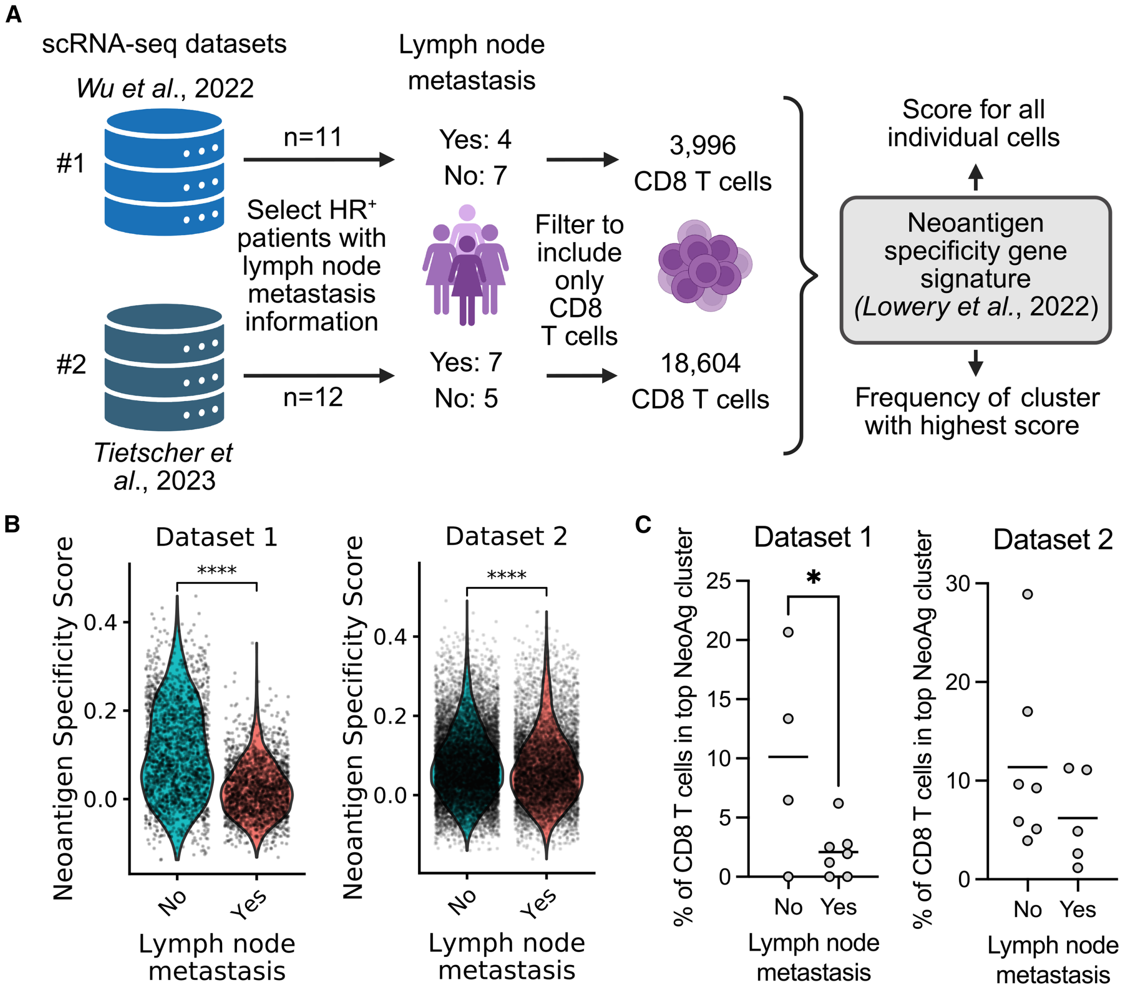

Figure 5. CD8 TILs from lymph node-positive HR+ breast cancer patients show reduced neoantigen-specific transcriptional signature in two independent scRNA-seq datasets

(A) Schematic of the analysis of the scRNA-seq datasets. The CD8 T cells from HR+ breast cancer patients were selected and submitted to a neoantigen-specific gene module analysis.

(B) Neoantigen-specific score of each individual CD8 T cell from patients with and without lymph node analysis. Dataset 1: yes n = 1,995; no n = 2,001; $p < 2.22e-16$; dataset 2: yes n = 6,944; no n = 11,660; p = 6.1e−14.

(C) Percentage of CD8 T cells that occupy the cluster with the highest neoantigen specificity score in patients with and without lymph node metastasis. Dataset 1: cluster LAG3; yes n = 7; no n = 4; p = 0.0421; dataset 2: cluster 3; yes n = 5; no n = 7; p = 0.2658. Two-tailed unpaired t test. ***$p < 0.001$, **$p < 0.01$, *$p < 0.05$.

specific transcriptional profiles that can be used to estimate the likelihood of neoantigen specificity.[25–27] We applied a CD8 neoantigen specificity transcriptional module score that was calculated using samples from different cancers types, including breast tumors.[26]

In both datasets, the CD8 TILs from lymph node-negative patients showed a significantly higher neoantigen specificity score compared to CD8 TILs from patients with lymph node metastasis (Dataset 1: $p < 2.22 \times 10^{-16}$; Dataset 2: $p = 6.1 \times 10^{-14}$; Figure 5B). The frequency of total CD8 T cells in the tumor was not different between patients with and without lymph node metastasis (Figure S7A). To estimate the frequency of neoantigen-specific CD8 T cells in each patient, we determined the cluster with the highest mean neoantigen specificity score and calculated its frequency across patients. The cluster with the highest module score in each dataset exhibited elevated expression of markers commonly associated with neoantigen specificity, such as CXCL13, *ENTPD1* (encodes CD39), and *ITGAE* (encodes CD103)[25–29] (Figures S7B–S7F). The frequency of the identified cluster was significantly lower in patients with lymph node metastasis in the first dataset (p = 0.0421; Figure 5C). A similar trend was uncovered in the second dataset, although the difference did not reach statistical significance (p = 0.2659). This may be explained by the design of the second dataset, which included an equal number of patients with exhausted and non-exhausted transcriptional signatures.[23] As some markers enriched in neoantigen-specific T cells, such as LAG3 and PDCD1 (encodes PD-1), are also associated with T cell

exhaustion,[26,27,30] this sampling strategy may have introduced a bias that obscured the relationship between lymph node status and the abundance of neoantigen-specific CD8 T cells.

The analysis of the two independent scRNA-seq datasets indicates that patients with lymph node metastasis may have lower frequency of neoantigen-specific CD8 T cells, which corroborates the previous results obtained when evaluating the tumor-reactive TILs using the tumor lysate.

DISCUSSION

In this study, we set out to dissect the tumor-reactive CD8 T cell response in HR+ breast cancer patients. We used autologous moDCs loaded with autologous tumor lysate to stimulate peripheral blood-derived T cells to investigate the overall circulating tumor-reactive T cell repertoire. We observed that the majority of early-stage HR+ breast cancer patients had a detectable tumor-reactive CD8 T cell response. Our study also provided a deep TCR profiling of tumor-reactive T cells in breast cancer, highlighting the diversity of the TCR repertoire in patients with a detectable antitumor response. Finally, we showed that the presence of circulating tumor-reactive CD8 T cells correlates with overall CD8 TIL infiltration and that circulating tumor-reactive CD8 T cell clones could also be found inside the tumor tissue.

By using an antigen-agnostic approach to evaluate the tumor-reactive T cell response, we avoided the challenge of selecting relevant tumor antigens. Previous studies have shown that breast cancer has lower CTA expression compared to other tumor types and that HR+ subtype tumors show an even more restricted CTA expression.[5,31–33] In this study, we tested our tumor-reactive CD8 T cells against 10 relevant tumor-associated antigens. We found responses to only two CTA (ACTL8 and PLAC1) in 20% of the patients. PLAC1-specific T cells with the ability to kill breast tumor lines were previously found in healthy donors.[34] Here, we have shown that breast cancer patients can naturally mount a response against these two proteins. T cells specific for other tumor antigens have been described in breast cancer,[14,15,17] but they are also only found in a small percentage of patients. A study of 20 breast cancer patients, for example, failed to identify T cells specific for three CTA,[16] further highlighting the difficulty of finding T cells specific for selected breast tumor antigens.

Since we used autologous tumor lysate as the antigen source to determine the tumor-specific response, we could, potentially, detect T cells specific to all tumor antigens that can be naturally processed by antigen-presenting cells simultaneously. With this broad approach, we were able to confirm our previous observation that most HR+ breast cancer patients have a detectable circulating antitumor CD8 T cell response.[9] We also uncovered that patients with a detectable antitumor response had a very diverse tumor-reactive CD8 TCR repertoire. However, although quite diverse, we found that tumor-reactive CD8 TCRs were mainly private, even among patients with shared human leukocyte antigen (HLA) alleles. This is consistent with the idea that each patient might be responding to a distinct set of tumor antigens, which could explain the difficulty in finding T cells specific to the same antigen in a high proportion of the patients. A recent proteomics study has found fewer shared CTAs in HR+ breast cancer patients compared to TNBC, further supporting this idea.[31] T cells specific for the same antigen can share TCR motifs, as has been previously shown for melanoma.[35] The fact that our TCR clustering analysis did not reveal many similar TCR sequences across patients reinforces the notion of highly individualized immune responses.

To evaluate the presence of tumor-reactive TIL, we matched TCR sequences from circulating tumor-reactive T cells with the TILs in the same donor. This approach enables us to identify the tumor-specific cells inside the tumor, regardless of their functionality. This is particularly important when considering that TILs tend to have an exhausted phenotype and may not respond to functional assays.[23,36,37]

We have also confirmed the results of the TIL specificity in two independent scRNA-seq datasets, using a different approach that did not rely on the detection of circulating tumor-reactive T cells. The transcriptional signature of the CD8 T cells was used to estimate the likelihood of neoantigen specificity. It is important to note that neoantigen specificity is associated with exhaustion,[26,27,30] which could confound interpretation. Nonetheless, given the private nature of the tumor-reactive TCR repertoire, this approach provided a practical way of validating our findings in available cohorts.

The histological subtypes of breast cancer are known to differ in biological and clinical behavior.[38,39] Transcriptional pathways related to immune response were previously shown to be enriched in lobular cancers compared to ductal ones.[40] Yet, lobular cancer has been described to have fewer TIL.[41] In our study, only ductal breast tumors showed extensive CD8 T cell infiltration. Another immune landscape analysis between ductal and lobular HR+ breast cancer showed remarkable similarities on the T cell compartment, with the biggest differences being found among the macrophages.[42] Our study advances this by showing no significant difference in the antitumor response when comparing patients with different histological subtypes.

Estrogen deprivation treatments show many immune-mediated effects.[43] Here, we did not find any significant impact of letrozole on the tumor-reactive T cell response, but only very few patients in our cohort received treatment. We have observed significant differences in the antitumoral responses of patients with early primary breast cancer and those with regional node metastases tumors, independently of their histological subtype.

The majority of patients with lymph node metastasis did not exhibit a measurable circulating antitumor CD8 T cell response. Among those who did, the proportion of tumor-reactive TILs was notably lower compared to early-stage patients. In contrast, the vast majority of our early-stage patients had a diverse circulating CD8 T cell response, capable of infiltrating the tumor. Although lymph node metastasis shows a positive correlation with tumor burden in HR+ breast cancer patients,[44] we did not find a significant difference in tumor size and tumor grade when comparing patients with a detectable or undetectable circulating antitumor CD8 T cell response. The same was true for other possible confounding variables such us age and treatment status. This indicates that lymph node metastasis is the key factor contributing to the diminished circulating antitumor CD8 T cell response in these patients.

CellPress
OPEN ACCESS

Tumor progression has been linked to changes in immune responses.[45] In advanced breast cancer patients, T cells specific for tumor-associated antigens have been detected only in the blood of patients that did not have circulating tumor cells.[14] The association between tumor progression and impaired antitumor immunity raises the question of causality and, if such a relationship exists, which event precedes the other. On one hand, a deficient T cell response might allow for early metastatic spread to lymph nodes. We found no difference in the tumor antigen load between patients with and without lymph node metastasis, indicating that the absence of circulating tumor-reactive T cells was not due to a lack of tumor antigens. Studies in melanoma have shown that tumor-specific CD8 T cells in lymph nodes can protect the lymph node against tumor seeding,[46] indicating that the presence of an antitumor response may help prevent nodal metastasis. On the other hand, the presence of tumor cells in lymph nodes could suppress the generation of tumor-reactive T cells. In HR^+ breast cancer patients, regulatory T cells (Treg) have been shown to accumulate in tumor-invaded lymph nodes, potentially impairing the generation of antitumor responses in these lymph nodes.[47] An increase in Foxp3 expression has been observed even in sentinel lymph nodes of breast cancer patients with molecular micro metastasis, indicating that Treg accumulation happens early during lymph node colonization.[48] These observations point to plausible mechanisms linking lymph node metastasis and impaired antitumor immunity, but the directionality of this interaction remains an open question that warrants further investigation.

Although we cannot determine if there is, indeed, a causal relation between lymph node metastasis and lack of an efficient antitumor response, we can infer that lymph node-positive HR^+ breast cancer patients might benefit from treatments aiming to improve their antitumor immunity. These could consist of vaccination strategies intended to generate a robust tumor-specific T cell response *in vivo,*[10,49,50] or by injecting preformed tumor-specific T cells, generated by transducing a recombinant TCR (TCR-T)[51–54] or a chimeric antigen receptor into T cells.[55–57] These strategies, though, rely on determining TCRs and antigens that can be used on many patients, which can be a challenge as mentioned before. The strategy used in this paper has the capacity to determine TCRs with clinical potential and, when associated with TCR deorphanization strategies,[58] can determine antigens of interest. For example, we were able to find a T cell clone (Br23 clone 3) that can recognize and kill multiple HLA*A02:01$^+$ tumor cell lines. This clone may be reacting to an antigen shared between all cell lines, possibly a CTA, or could be cross-reactive to different antigens being expressed by each line.[59] Regardless, by being able to recognize and kill multiple cancer cell lines, this clone shows great potential to be utilized for treatment. Extending the clonal analysis to a larger cohort of patients could support the identification of new immunotherapeutic targets for HR^+ breast cancer.

The reduction in tumor-reactive TILs that was observed in patients with lymph node involvement may help explain the difference in efficacy of ICI treatment across different stages of HR^+ breast cancer. While limited efficacy was noted when treating advanced HR^+ breast cancer, studies in early-stage patients have shown that a subset of these patients might benefit from ICI treatment.[60] Determining predictive indicators of response is integral for selecting the patients that could benefit the most from this treatment, and many immune-related markers are currently under investigation.[61] The role of the antitumor T cell response as a biomarker for ICI response in HR^+ breast cancer is a topic that merits further investigation.

In conclusion, we were able to do an in-depth analysis of the antitumor CD8 T cell repertoire in HR^+ patients. We found that early-stage patients have a diverse antitumor response, specific to multiple antigens and with the ability to infiltrate the tumor. The tumor-reactive T cells found in these patients could be mobilized to treat breast cancer patients with lymph node metastasis, since they had an impaired antitumoral response. Our results add to the explanation of why HR^+ breast cancer patients benefit less from checkpoint blockage treatments compared to TNBC patients but also open the avenue to explore other immunotherapeutic interventions, such as the use of vaccines and TCR-T, that might provide greater clinical benefit.

Limitations of the study

We recognize that the present study presents some limitations, for example, with the use of the TCR approach to detect tumor-reactive TIL. The high diversity of the blood T cell repertoire compared to the tumor may result in reduced detection of rare circulating tumor-reactive T cells that are also present within TILs. To minimize this problem, we stimulated at least 1 million blood T cells with tumor lysate. Furthermore, to have sufficient cells for the TCR repertoire analysis, we have relied on the expansion of CD8 T cells with feeders, which could lead to a skewing of TCR diversity. We have confirmed the expansion of CD8 T cell clones with different CD25 and CFSE levels after coculture with DC, reassuring that the repertoire diversity could be captured after feeder expansion. It is also important to note the overall small number of CD8 TCRs obtained from the TIL. This is likely a result from a combination of limited T cell infiltration in breast cancer and the restriction of tissue availability from small tumors, often increasing the difficulty of performing this type of study. Despite the low numbers, we successfully mapped at least one tumor-reactive CD8 TCR in the TIL in all but one patient.

RESOURCE AVAILABILITY

Lead contact

Requests for further information and resources should be directed to and will be fulfilled by the lead contact, Tao Dong (tao.dong@ndm.ox.ac.uk).

Materials availability

This study did not generate new unique reagents.

Data and code availability

- All data reported in this paper will be shared by the lead contact upon request.
- This paper analyzes existing, publicly available data. TRON is accessible at https://doi.org/10.1186/s13073-015-0240-5 and TCGA is accessible using the RTCGA package in R. The scRNA-seq datasets are accessible from the GEO series accession number GEO: GSE176078[24] and the ArrayExpress database at EMBL-EBI under accession number ArrayExpress: E-MTAB-10607.[23]
- This paper does not report original code.

- Any additional information required to reanalyze the data reported in this paper is available from the lead contact upon request.

ACKNOWLEDGMENTS

This work was supported by the Chinese Academy of Medical Sciences (CAMS) Innovation Fund for Medical Science (CIFMS), China (grant number: 2024-I2M-2-001-1) (T.D., M.P.P., E.A, B.S., T.S., F.G., X.Y., D.M.-P., A.B., R.A.F., Y.P., and M.H.B.A.H.), and UK Medical Research Council (grant number: MR/Y015347/1) (T.D., M.P.P., and Y.P.). We thank all patients who volunteered to participate in this study. We express our gratitude to all members of Tao Dong's lab for providing their insights on the work. We thank Francesca Buffa and Helen Sheldon for providing the established breast cancer lines. We thank Christine Jesus, Sorayya Moradi, and Natalia Perdek (ORB, Nuffield Department of Surgical Sciences, University of Oxford) for assisting in patient acquisition and sampling. We acknowledge the contribution to this study made by the Oxford Centre for Histopathology Research and the Oxford Radcliffe Biobank, which are supported by the University of Oxford, the Oxford CRUK Cancer Centre, and the NIHR Oxford Biomedical Research Centre (Molecular Diagnostics Theme/Multimodal Pathology Subtheme), and the NIHR CRN Thames Valley Network. The views expressed are those of the author(s) and not necessarily of the NHS; the NIHR; or the Department of Health, U.K. Figures 1A, 4D, and 5A were created with BioRender.com.

AUTHOR CONTRIBUTIONS

T.D., M.P.P., and A.A. conceptualized the study; M.P.P. and T.D. designed the experiments; T.D. acquired the main funding; M.P.P., T.D., and Y.P. supervised the data analysis and experiments; M.P.P. performed most of the experiments and data analysis; Y.P. provided key technical support and advice; E.A., T.S., B.S., F.G., X.Y., L.C., A.B., L.W., and N.K.A. assisted in experiments and data analysis; A.L.H., S.-A.C., C.W., P.S., A.B., M.H.B.A.H., R.A.F., S.R.L., and C.V. provided technical assistance and critical reagents; A.A., T.D., M.P.P., and C.C. coordinated the clinical collaborations; D.M.-P., R.T., and E.W. collected clinical samples and clinical data; T.R. performed HLA typing and next-generation sequencing; A.A. identified, selected, and took consent of the patients; M.P.P. wrote the original draft; T.D., A.L.H., Y.P., B.S., E.A., M.H.B.A.H., A.B., and S.R.L. reviewed and edited the manuscript and figures.

DECLARATION OF INTERESTS

The authors declare no competing interests.

STAR★METHODS

Detailed methods are provided in the online version of this paper and include the following:

- KEY RESOURCES TABLE
- EXPERIMENTAL MODEL AND STUDY PARTICIPANT DETAILS
    - Human participants
    - Breast cancer cell lines
- METHOD DETAILS
    - Tissue processing and lysate preparation
    - Blood processing, T cell isolation and monocyte-derived dendritic cells differentiation
    - Proliferation assay and isolation of lysate-specific T cells
    - Generation of tumor-reactive T cell lines and T cell clones
    - Evaluation of T cell response against tumor cell lines and CTA peptide pools
    - Killing assay
    - Generation of CD8 TIL cell lines for TCR sequencing
    - Deep sequencing of the TCR of T cell lines and clones
    - Immunohistochemistry analysis
    - Tumor-reactive TCR repertoire analysis
    - Determination of frequency of tumor-reactive and pathogen-specific TRB in tumor
    - Analysis of expression of tumor antigens on the breast cancer cell lines
    - Analysis of expression of tumor antigens on cancer patients
    - Single-cell RNA sequencing data acquisition and analysis
- QUANTIFICATION AND STATISTICAL ANALYSIS
    - Statistical analysis

SUPPLEMENTAL INFORMATION

Supplemental information can be found online at https://doi.org/10.1016/j.xcrm.2025.102252.

Received: January 21, 2025
Revised: April 22, 2025
Accepted: July 1, 2025
Published: July 28, 2025

REFERENCES

1. Bray, F., Laversanne, M., Sung, H., Ferlay, J., Siegel, R.L., Soerjomataram, I., and Jemal, A. (2024). Global cancer statistics 2022: GLOBOCAN estimates of incidence and mortality worldwide for 36 cancers in 185 countries. CA Cancer J. Clin. *74*, 229–263. https://doi.org/10.3322/CAAC.21834.
2. Wang, K., Yang, J., Wang, B., Liu, Q., Wang, X., Yin, Y., Wang, H., Wang, S., Hao, C., Hao, X., et al. (2024). Expert consensus on the clinical application of immunotherapy in breast cancer: 2024. Transl. Breast Cancer Res. *5*, 9. https://doi.org/10.21037/TBCR-24-15.
3. Raskov, H., Orhan, A., Christensen, J.P., and Gögenur, I. (2021). Cytotoxic CD8+ T cells in cancer and cancer immunotherapy. Br. J. Cancer, 359–359367. https://doi.org/10.1038/s41416-020-01048-4.
4. Semiglazov, V., Tseluiko, A., Kudaybergenova, A., Artemyeva, A., Krivorotko, P., and Donskih, R. (2022). Immunology and immunotherapy in breast cancer. Cancer Biol. Med. *19*, 609–618. https://doi.org/10.20892/J.ISSN.2095-3941.2021.0597.
5. Yao, J., Caballero, O.L., Yung, W.K.A., Weinstein, J.N., Riggins, G.J., Strausberg, R.L., and Zhao, Q. (2014). Tumor subtype-specific cancer-testis antigens as potential biomarkers and immunotherapeutic targets for cancers. Cancer Immunol. Res. *2*, 371–379. https://doi.org/10.1158/2326-6066.CIR-13-0088.
6. Narang, P., Chen, M., Sharma, A.A., Anderson, K.S., and Wilson, M.A. (2019). The neoepitope landscape of breast cancer: Implications for immunotherapy. BMC Cancer *19*, 200–210. https://doi.org/10.1186/S12885-019-5402-1.
7. Schumacher, T.N., and Schreiber, R.D. (2015). Neoantigens in cancer immunotherapy. Science *348*, 69–74. https://doi.org/10.1126/SCIENCE.AAA4971.
8. Thorsson, V., Gibbs, D.L., Brown, S.D., Wolf, D., Bortone, D.S., Ou Yang, T.-H., Porta-Pardo, E., Gao, G.F., Plaisier, C.L., Eddy, J.A., et al. (2018). The Immune Landscape of Cancer. Immunity *48*, 812–830.e14. https://doi.org/10.1016/j.immuni.2018.03.023.
9. Pinho, M.P., Patente, T.A., Flatow, E.A., Sallusto, F., and Barbuto, J.A.M. (2019). Frequency determination of breast tumor-reactive CD4 and CD8 T cells in humans: unveiling the antitumor immune response. OncoImmunology *8*, 1607674. https://doi.org/10.1080/2162402X.2019.1607674.
10. Zacharakis, N., Huq, L.M., Seitter, S.J., Kim, S.P., Gartner, J.J., Sindiri, S., Hill, V.K., Li, Y.F., Paria, B.C., Ray, S., et al. (2022). Breast Cancers Are Immunogenic: Immunologic Analyses and a Phase II Pilot Clinical Trial Using Mutation-Reactive Autologous Lymphocytes. J. Clin. Oncol. *40*, 1741–1754. https://doi.org/10.1200/JCO.21.02170.
11. Morisaki, T., Kubo, M., Umebayashi, M., Yew, P.Y., Yoshimura, S., Park, J. H., Kiyotani, K., Kai, M., Yamada, M., Oda, Y., et al. (2021). Neoantigens elicit T cell responses in breast cancer. Sci. Rep. *11*, 13590. https://doi.org/10.1038/S41598-021-91358-1.

CellPress
OPEN ACCESS

12. Lloyd, M.R., Jhaveri, K., Kalinsky, K., Bardia, A., and Wander, S.A. (2024). Precision therapeutics and emerging strategies for HR-positive metastatic breast cancer. Nat. Rev. Clin. Oncol., 743–743761. https://doi.org/10.1038/s41571-024-00935-6.

13. Goldberg, J., Pastorello, R.G., Vallius, T., Davis, J., Cui, Y.X., Agudo, J., Waks, A.G., Keenan, T., McAllister, S.S., Tolaney, S.M., et al. (2021). The Immunology of Hormone Receptor Positive Breast Cancer. Front. Immunol. *12*, 674192. https://doi.org/10.3389/FIMMU.2021.674192.

14. Muraro, E., Del Ben, F., Turetta, M., Cesselli, D., Bulfoni, M., Zamarchi, R., Rossi, E., Spazzapan, S., Dolcetti, R., Steffan, A., and Brisotto, G. (2022). Clinical relevance of the combined analysis of circulating tumor cells and anti-tumor T-cell immunity in metastatic breast cancer patients. Front. Oncol. *12*, 983887. https://doi.org/10.3389/FONC.2022.983887.

15. Viborg, N., Ramskov, S., Andersen, R.S., Sturm, T., Fugmann, T., Bentzen, A.K., Rafa, V.M., Straten, P.T., Svane, I.M., Met, Ö., and Hadrup, S.R. (2019). T cell recognition of novel shared breast cancer antigens is frequently observed in peripheral blood of breast cancer patients. OncoImmunology *8*, e1663107. https://doi.org/10.1080/2162402X.2019.1663107.

16. Nagorsen, D., Scheibenbogen, C., Schaller, G., Leigh, B., Schmittel, A., Letsch, A., Thiel, E., and Keilholz, U. (2003). Differences in T-cell immunity toward tumor-associated antigens in colorectal cancer and breast cancer patients. Int. J. Cancer *105*, 221–225. https://doi.org/10.1002/IJC.11052.

17. Inokuma, M., dela Rosa, C., Schmitt, C., Haaland, P., Siebert, J., Petry, D., Tang, M., Suni, M.A., Ghanekar, S.A., Gladding, D., et al. (2007). Functional T cell responses to tumor antigens in breast cancer patients have a distinct phenotype and cytokine signature. J. Immunol. *179*, 2627–2633. https://doi.org/10.4049/JIMMUNOL.179.4.2627.

18. Koboldt, D.C., Fulton, R.S., McLellan, M.D., Schmidt, H., Kalicki-Veizer, J., McMichael, J.F., Fulton, L.L., Dooling, D.J., Ding, L., Mardis, E.R., et al. (2012). Comprehensive molecular portraits of human breast tumours. Nature *490*, 61–70. https://doi.org/10.1038/nature11412.

19. Scholtalbers, J., Boegel, S., Bukur, T., Byl, M., Goerges, S., Sorn, P., Loewer, M., Sahin, U., and Castle, J.C. (2015). TCLP: An online cancer cell line catalogue integrating HLA type, predicted neo-epitopes, virus and gene expression. Genome Med. *7*, 118. https://doi.org/10.1186/S13073-015-0240-5.

20. Stanton, S.E., Adams, S., and Disis, M.L. (2016). Variation in the Incidence and Magnitude of Tumor-Infiltrating Lymphocytes in Breast Cancer Subtypes: A Systematic Review. JAMA Oncol. *2*, 1354–1360. https://doi.org/10.1001/JAMAONCOL.2016.1061.

21. Miyan, M., Schmidt-Mende, J., Kiessling, R., Poschke, I., and de Boniface, J. (2016). Differential tumor infiltration by T-cells characterizes intrinsic molecular subtypes in breast cancer. J. Transl. Med. *14*, 227. https://doi.org/10.1186/S12967-016-0983-9.

22. Narvaez, D., Nadal, J., Nervo, A., Costanzo, M.V., Paletta, C., Petracci, F. E., Rivero, S., Ostinelli, A., Freile, B., Enrico, D., et al. (2024). The Emerging Role of Tertiary Lymphoid Structures in Breast Cancer: A Narrative Review. Cancers (Basel) *16*, 396. https://doi.org/10.3390/CANCERS16020396.

23. Tietscher, S., Wagner, J., Anzeneder, T., Langwieder, C., Rees, M., Sobottka, B., de Souza, N., and Bodenmiller, B. (2023). A comprehensive single-cell map of T cell exhaustion-associated immune environments in human breast cancer. Nat. Commun. *14*, 98. https://doi.org/10.1038/S41467-022-35238-W.

24. Wu, S.Z., Al-Eryani, G., Roden, D.L., Junankar, S., Harvey, K., Andersson, A., Thennavan, A., Wang, C., Torpy, J.R., Bartonicek, N., et al. (2021). A single-cell and spatially resolved atlas of human breast cancers. Nat. Genet. *53*, 1334–1347. https://doi.org/10.1038/S41588-021-00911-1.

25. Zheng, C., Fass, J.N., Shih, Y.P., Gunderson, A.J., Sanjuan Silva, N., Huang, H., Bernard, B.M., Rajamanickam, V., Slagel, J., Bifulco, C.B., et al. (2022). Transcriptomic profiles of neoantigen-reactive T cells in human gastrointestinal cancers. Cancer Cell *40*, 410–423.e7. https://doi.org/10.1016/j.ccell.2022.03.005.

26. Lowery, F.J., Krishna, S., Yossef, R., Parikh, N.B., Chatani, P.D., Zacharakis, N., Parkhurst, M.R., Levin, N., Sindiri, S., Sachs, A., et al. (2022). Molecular signatures of antitumor neoantigen-reactive T cells from metastatic human cancers. Science *375*, 877–884. https://doi.org/10.1126/SCIENCE.ABL5447.

27. Hanada, K.I., Zhao, C., Gil-Hoyos, R., Gartner, J.J., Chow-Parmer, C., Lowery, F.J., Krishna, S., Prickett, T.D., Kivitz, S., Parkhurst, M.R., et al. (2022). A phenotypic signature that identifies neoantigen-reactive T cells in fresh human lung cancers. Cancer Cell *40*, 479–493.e6. https://doi.org/10.1016/J.CCELL.2022.03.012.

28. Duhen, T., Duhen, R., Montler, R., Moses, J., Moudgil, T., De Miranda, N. F., Goodall, C.P., Blair, T.C., Fox, B.A., McDermott, J.E., et al. (2018). Co-expression of CD39 and CD103 identifies tumor-reactive CD8 T cells in human solid tumors. Nat. Commun. *9*, 2724. https://doi.org/10.1038/s41467-018-05072-0.

29. Simoni, Y., Becht, E., Fehlings, M., Loh, C.Y., Koo, S.L., Teng, K.W.W., Yeong, J.P.S., Nahar, R., Zhang, T., Kared, H., et al. (2018). Bystander CD8+ T cells are abundant and phenotypically distinct in human tumour infiltrates. Nature *557*, 575–579. https://doi.org/10.1038/s41586-018-0130-2.

30. Gros, A., Robbins, P.F., Yao, X., Li, Y.F., Turcotte, S., Tran, E., Wunderlich, J.R., Mixon, A., Farid, S., Dudley, M.E., et al. (2014). PD-1 identifies the patient-specific CD8+ tumor-reactive repertoire infiltrating human tumors. J. Clin. Investig. *124*, 2246–2259. https://doi.org/10.1172/JCI73639.

31. Kina, E., Laverdure, J.P., Durette, C., Lanoix, J., Courcelles, M., Zhao, Q., Apavaloaei, A., Larouche, J.D., Hardy, M.P., Vincent, K., et al. (2024). Breast cancer immunopeptidomes contain numerous shared tumor antigens. J. Clin. Investig. *134*, e166740. https://doi.org/10.1172/JCI166740.

32. Curigliano, G., Bagnardi, V., Ghioni, M., Louahed, J., Brichard, V., Lehmann, F.F., Marra, A., Trapani, D., Criscitiello, C., and Viale, G. (2020). Expression of tumor-associated antigens in breast cancer subtypes. Breast *49*, 202–209. https://doi.org/10.1016/J.BREAST.2019.12.002.

33. Grigoriadis, A., Caballero, O.L., Hoek, K.S., Da Silva, L., Chen, Y.T., Shin, S.J., Jungbluth, A.A., Miller, L.D., Clouston, D., Cebon, J., et al. (2009). CT-X antigen expression in human breast cancer. Proc. Natl. Acad. Sci. USA *106*, 13493–13498. https://doi.org/10.1073/PNAS.0906840106.

34. Liu, W., Zhai, M., Wu, Z., Qi, Y., Wu, Y., Dai, C., Sun, M., Li, L., and Gao, Y. (2012). Identification of a novel HLA-A2-restricted cytotoxic T lymphocyte epitope from cancer-testis antigen PLAC1 in breast cancer. Amino Acids *42*, 2257–2265. https://doi.org/10.1007/S00726-011-0966-3.

35. Huuhtanen, J., Chen, L., Jokinen, E., Kasanen, H., Lönnberg, T., Kreutzman, A., Peltola, K., Hernberg, M., Wang, C., Yee, C., et al. (2022). Evolution and modulation of antigen-specific T cell responses in melanoma patients. Nat. Commun. *13*, 5988. https://doi.org/10.1038/s41467-022-33720-z.

36. Egelston, C.A., Guo, W., Tan, J., Avalos, C., Simons, D.L., Lim, M.H., Huang, Y.J., Nelson, M.S., Chowdhury, A., Schmolze, D.B., et al. (2022). Tumor-infiltrating exhausted CD8+ T cells dictate reduced survival in premenopausal estrogen receptor–positive breast cancer. JCI Insight *7*, e153963. https://doi.org/10.1172/JCI.INSIGHT.153963.

37. Jiang, Y., Li, Y., and Zhu, B. (2015). T-cell exhaustion in the tumor microenvironment. Cell Death Dis. *6*, e1792. https://doi.org/10.1038/cddis.2015.162.

38. Weigelt, B., Geyer, F.C., and Reis-Filho, J.S. (2010). Histological types of breast cancer: How special are they? Mol. Oncol. *4*, 192–208. https://doi.org/10.1016/J.MOLONC.2010.04.004.

39. Barroso-Sousa, R., and Metzger-Filho, O. (2016). Differences between invasive lobular and invasive ductal carcinoma of the breast: results and therapeutic implications. Ther. Adv. Med. Oncol. *8*, 261–266. https://doi.org/10.1177/1758834016644156.

40. Du, T., Zhu, L., Levine, K.M., Tasdemir, N., Lee, A.V., Vignali, D.A.A., Houten, B.V., Tseng, G.C., and Oesterreich, S. (2018). Invasive lobular and ductal breast carcinoma differ in immune response, protein translation

efficiency and metabolism. Sci. Rep. *8*, 7205. https://doi.org/10.1038/S41598-018-25357-0.

41. Desmedt, C., Salgado, R., Fornili, M., Pruneri, G., Van Den Eynden, G., Zoppoli, G., Rothé, F., Buisseret, L., Garaud, S., Willard-Gallo, K., et al. (2018). Immune Infiltration in Invasive Lobular Breast Cancer. J. Natl. Cancer Inst. *110*, 768–776. https://doi.org/10.1093/JNCI/DJX268.

42. Onkar, S., Cui, J., Zou, J., Cardello, C., Cillo, A.R., Uddin, M.R., Sagan, A., Joy, M., Osmanbeyoglu, H.U., Pogue-Geile, K.L., et al. (2023). Immune landscape in invasive ductal and lobular breast cancer reveals a divergent macrophage-driven microenvironment. Nat. Cancer *4*, 516–534. https://doi.org/10.1038/s43018-023-00527-w.

43. McGuinness, C., and Britt, K.L. (2024). Estrogen receptor regulation of the immune microenvironment in breast cancer. J. Steroid Biochem. Mol. Biol. *240*, 106517. https://doi.org/10.1016/J.JSBMB.2024.106517.

44. Min, S.K., Lee, S.K., Woo, J., Jung, S.M., Ryu, J.M., Yu, J., Lee, J.E., Kim, S.W., Chae, B.J., and Nam, S.J. (2021). Relation Between Tumor Size and Lymph Node Metastasis According to Subtypes of Breast Cancer. J. Breast Cancer *24*, 75–84. https://doi.org/10.4048/JBC.2021.24.E4.

45. de Visser, K.E., and Joyce, J.A. (2023). The evolving tumor microenvironment: From cancer initiation to metastatic outgrowth. Cancer Cell *41*, 374–403. https://doi.org/10.1016/J.CCELL.2023.02.016.

46. Molodtsov, A.K., Khatwani, N., Vella, J.L., Lewis, K.A., Zhao, Y., Han, J., Sullivan, D.E., Searles, T.G., Preiss, N.K., Shabaneh, T.B., et al. (2021). Resident memory CD8+ T cells in regional lymph nodes mediate immunity to metastatic melanoma. Immunity *54*, 2117–2132.e7. https://doi.org/10.1016/J.IMMUNI.2021.08.019.

47. Núñez, N.G., Tosello Boari, J., Ramos, R.N., Richer, W., Cagnard, N., Anderfuhren, C.D., Niborski, L.L., Bigot, J., Meseure, D., De La Rochere, P., et al. (2020). Tumor invasion in draining lymph nodes is associated with Treg accumulation in breast cancer patients. Nat. Commun. *11*, 1–15. https://doi.org/10.1038/s41467-020-17046-2.

48. Nakamura, R., Sakakibara, M., Nagashima, T., Sangai, T., Arai, M., Fujimori, T., Takano, S., Shida, T., Nakatani, Y., and Miyazaki, M. (2009). Accumulation of regulatory T cells in sentinel lymph nodes is a prognostic predictor in patients with node-negative breast cancer. Eur. J. Cancer *45*, 2123–2131. https://doi.org/10.1016/J.EJCA.2009.03.024.

49. Saxena, M., van der Burg, S.H., Melief, C.J.M., and Bhardwaj, N. (2021). Therapeutic cancer vaccines. Nat. Rev. Cancer *21*, 360–378. https://doi.org/10.1038/s41568-021-00346-0.

50. Al-Hawary, S.I.S., Saleh, E.A.M., Mamajanov, N.A., S Gilmanova, N., Alsaab, H.O., Alghamdi, A., Ansari, S.A., Alawady, A.H.R., Alsaalamy, A. H., and Ibrahim, A.J. (2023). Breast cancer vaccines; A comprehensive and updated review. Pathol. Res. Pract. *249*, 154735. https://doi.org/10.1016/J.PRP.2023.154735.

51. Klebanoff, C.A., Chandran, S.S., Baker, B.M., Quezada, S.A., and Ribas, A. (2023). T cell receptor therapeutics: immunological targeting of the intracellular cancer proteome. Nat. Rev. Drug Discov. *22*, 996–1017. https://doi.org/10.1038/s41573-023-00809-z.

52. Chamorro, D.F., Somes, L.K., and Hoyos, V. (2023). Engineered Adoptive T-Cell Therapies for Breast Cancer: Current Progress, Challenges, and Potential. Cancers (Basel) *16*, 124. https://doi.org/10.3390/CANCERS16010124/S1.

53. Kortleve, D., Hammerl, D., van Brakel, M., Wijers, R., Roelofs, D., Kroese, K., Timmermans, M.M., Liao, C.-Y., Huang, S., Trapman-Jansen, A., et al. (2024). TCR-Engineered T Cells Directed against Ropporin-1 Constitute a Safe and Effective Treatment for Triple-Negative Breast Cancer. Cancer Discov. *14*, 2450–2470. https://doi.org/10.1158/2159-8290.CD-24-0168.

54. Li, Q., Liu, M., Wu, M., Zhou, X., Wang, S., Hu, Y., Wang, Y., He, Y., Zeng, X., Chen, J., et al. (2018). PLAC1-specific TCR-engineered T cells mediate antigen-specific antitumor effects in breast cancer. Oncol. Lett. *15*, 5924–5932. https://doi.org/10.3892/ol.2018.8075.

55. Dagar, G., Gupta, A., Masoodi, T., Nisar, S., Merhi, M., Hashem, S., Chauhan, R., Dagar, M., Mirza, S., Bagga, P., et al. (2023). Harnessing the potential of CAR-T cell therapy: progress, challenges, and future directions in hematological and solid tumor treatments. J. Transl. Med. *21*, 449. https://doi.org/10.1186/S12967-023-04292-3.

56. Buono, G., Capozzi, M., Caputo, R., Lauro, V.D., Cianniello, D., Piezzo, M., Cocco, S., Martinelli, C., Verrazzo, A., Tafuro, M., et al. (2025). CAR-T cell therapy for breast cancer: Current status and future perspective. Cancer Treat Rev. *133*, 102868. https://doi.org/10.1016/J.CTRV.2024.102868.

57. Niu, Z., Wu, J., Zhao, Q., Zhang, J., Zhang, P., and Yang, Y. (2024). CAR-based immunotherapy for breast cancer: peculiarities, ongoing investigations, and future strategies. Front. Immunol. *15*, 1385571. https://doi.org/10.3389/FIMMU.2024.1385571.

58. Gee, M.H., Han, A., Lofgren, S.M., Beausang, J.F., Mendoza, J.L., Birnbaum, M.E., Bethune, M.T., Fischer, S., Yang, X., Gomez-Eerland, R., et al. (2018). Antigen Identification for Orphan T Cell Receptors Expressed on Tumor-Infiltrating Lymphocytes. Cell *172*, 549–563.e16. https://doi.org/10.1016/J.CELL.2017.11.043.

59. Dolton, G., Rius, C., Wall, A., Szomolay, B., Bianchi, V., Galloway, S.A.E., Hasan, M.S., Morin, T., Caillaud, M.E., Thomas, H.L., et al. (2023). Targeting of multiple tumor-associated antigens by individual T cell receptors during successful cancer immunotherapy. Cell *186*, 3333–3349.e27. https://doi.org/10.1016/J.CELL.2023.06.020.

60. Schlam, I., Corti, C., Sammons, S., Mittendorf, E.A., and Tolaney, S.M. (2024). Checkpoint inhibition for early-stage hormone receptor-positive breast cancer. Expert Opin. Biol. Ther. *24*, 511–520. https://doi.org/10.1080/14712598.2024.2370395.

61. Zagami, P., Cortés, J., Carey, L.A., and Curigliano, G. (2024). Immunotherapy in the treatment landscape of hormone receptor-positive (HR+) early breast cancer: is new data clinical practice changing? ESMO Open *9*, 103695. https://doi.org/10.1016/j.esmoop.2024.103695.

62. Shugay, M., Bagaev, D.V., Zvyagin, I.V., Vroomans, R.M., Crawford, J.C., Dolton, G., Komech, E.A., Sycheva, A.L., Koneva, A.E., Egorov, E.S., et al. (2018). VDJdb: a curated database of T-cell receptor sequences with known antigen specificity. Nucleic Acids Res. *46*, D419–D427. https://doi.org/10.1093/NAR/GKX760.

63. Bolotin, D.A., Poslavsky, S., Mitrophanov, I., Shugay, M., Mamedov, I.Z., Putintseva, E.V., and Chudakov, D.M. (2015). MiXCR: software for comprehensive adaptive immunity profiling. Nat. Methods *12*, 380–381. https://doi.org/10.1038/nmeth.3364.

64. ImmunoMind Team (2019). immunarch: An R Package for Painless Bioinformatics Analysis of T-Cell and B-Cell Immune Repertoires. Preprint at Zenodo. https://doi.org/10.5281/zenodo.3367200.

65. Gu, Z., Gu, L., Eils, R., Schlesner, M., and Brors, B. (2014). circlize implements and enhances circular visualization in R. Bioinformatics *30*, 2811–2812. https://doi.org/10.1093/BIOINFORMATICS/BTU393.

66. Hetzel, J.-P., Saeedghalati, M. cre, Farahpour, F., and Hoffmann, D. (2023). turboGliph: Find Specificity Groups with GLIPH and GLIPH2 Method. https://github.com/HetzDra/turboGliph/blob/main/DESCRIPTION.

67. Wagih, O. (2017). ggseqlogo: a versatile R package for drawing sequence logos. Bioinformatics *33*, 3645–3647. https://doi.org/10.1093/BIOINFORMATICS/BTX469.

68. Wickham, H. (2016). ggplot2: Elegant Graphics for Data Analysis (Springer Science & Business Media).

69. Hao, Y., Stuart, T., Kowalski, M.H., Choudhary, S., Hoffman, P., Hartman, A., Srivastava, A., Molla, G., Madad, S., Fernandez-Granda, C., and Satija, R. (2024). Dictionary learning for integrative, multimodal and scalable single-cell analysis. Nat. Biotechnol. *42*, 293–304. https://doi.org/10.1038/S41587-023-01767-Y.

70. Huang, M., Wang, J., Torre, E., Dueck, H., Shaffer, S., Bonasio, R., Murray, J.I., Raj, A., Li, M., and Zhang, N.R. (2018). SAVER: gene expression

recovery for single-cell RNA sequencing. Nat. Methods *15*, 539–542. https://doi.org/10.1038/s41592-018-0033-z.

71. Pinho, M.P., Sundarasetty, B.S., Bergami-Santos, P.C., Steponavicius-Cruz, K., Ferreira, A.K., Stripecke, R., and Barbuto, J.A.M. (2016). Dendritic-tumor cell hybrids induce tumor-specific immune responses more effectively than the simple mixture of dendritic and tumor cells. Cytotherapy *18*, 570–580. https://doi.org/10.1016/j.jcyt.2016.01.005.

72. Pinho, M.P., Lepski, G.A., Rehder, R., Chauca-Torres, N.E., Evangelista, G.C.M., Teixeira, S.F., Flatow, E.A., de Oliveira, J.V., Fogolin, C.S., Peres, N., et al. (2022). Near-Complete Remission of Glioblastoma in a Patient Treated with an Allogenic Dendritic Cell-Based Vaccine: The Role of Tumor-Specific CD4+T-Cell Cytokine Secretion Pattern in Predicting Response and Recurrence. Int. J. Mol. Sci. *23*, 5396. https://doi.org/10.3390/IJMS23105396.

73. Hamid, M.H.B.A., Cespedes, P.F., Jin, C., Chen, J.L., Gileadi, U., Antoun, E., Liang, Z., Gao, F., Teague, R., Manoharan, N., et al. (2024). Unconventional human CD61 pairing with CD103 promotes TCR signaling and antigen-specific T cell cytotoxicity. Nat. Immunol. *25*, 834–846. https://doi.org/10.1038/s41590-024-01802-3.

74. Peng, Y., Felce, S.L., Dong, D., Penkava, F., Mentzer, A.J., Yao, X., Liu, G., Yin, Z., Chen, J.L., Lu, Y., et al. (2021). An immunodominant NP105–113-B*07:02 cytotoxic T cell response controls viral replication and is associated with less severe COVID-19 disease. Nat. Immunol. *23*, 50–61. https://doi.org/10.1038/s41590-021-01084-z.

75. Bankhead, P., Loughrey, M.B., Fernández, J.A., Dombrowski, Y., McArt, D.G., Dunne, P.D., McQuaid, S., Gray, R.T., Murray, L.J., Coleman, H.G., et al. (2017). QuPath: Open source software for digital pathology image analysis. Sci. Rep. *7*, 1–7. https://doi.org/10.1038/s41598-017-17204-5.

76. Almeida, L.G., Sakabe, N.J., de Oliveira, A.R., Silva, M.C.C., Mundstein, A.S., Cohen, T., Chen, Y.T., Chua, R., Gurung, S., Gnjatic, S., et al. (2009). CTdatabase: a knowledge-base of high-throughput and curated data on cancer-testis antigens. Nucleic Acids Res. *37*, D816–D819. https://doi.org/10.1093/NAR/GKN673.

77. Cheang, M.C.U., Martin, M., Nielsen, T.O., Prat, A., Voduc, D., Rodriguez-Lescure, A., Ruiz, A., Chia, S., Shepherd, L., Ruiz-Borrego, M., et al. (2015). Defining Breast Cancer Intrinsic Subtypes by Quantitative Receptor Expression. Oncologist *20*, 474–482. https://doi.org/10.1634/THEONCOLOGIST.2014-0372.

STAR★METHODS

KEY RESOURCES TABLE

REAGENT or RESOURCE	SOURCE	IDENTIFIER
**Antibodies**		
PerCPCy5.5 anti-human CD8 clone SK1	Biolegend	Cat# 344710; RRID:AB_2044010
BV650 anti-human CD4 clone SK3	BD Biosciences	Cat# 563875; RRID:AB_2744425
PE-CF594 anti-human CD25 clone M-A251	BD Biosciences	Cat# 562403; RRID:AB_11151919
APC anti-human ICOS clone C398.4A	Biolegend	Cat# 313510; RRID:AB_416334
BV605 anti-human CD107a clone H4A3	Biolegend	Cat# 328634; RRID:AB_2563851
AF700 anti-human CD4 clone RPA-T4	Biolegend	Cat# 300526; RRID:AB_493743
BUV737 anti-human IFN-γ clone 4S.B3	BD Biosciences	Cat# 612845; RRID:AB_2870167
PECy7 anti-human TNF-α clone MAb11	Biolegend	Cat# 563996; RRID:AB_2738533
APC-H7 anti-human MIP1β clone D21-1351	BD Biosciences	Cat# 561280; RRID:AB_10611567
Purified anti-human HLA-ABC clone W6/32	Biolegend	Cat# 311428; RRID:AB_2561492
Purified anti-human HLA-A2 clone BB7.2	Sigma	Cat# SAB4700298; RRID:AB_10897783
Ultra-LEAF^{TM} Purified Mouse IgG2a, κ Isotype Ctrl Antibody	Biolegend	Cat# 400264; RRID:AB_11148947
Ultra-LEAF^{TM} Purified Mouse IgG2b, κ Isotype Ctrl Antibody	Biolegend	Cat# 401215; RRID:AB_3097073
**Biological samples**		
Human Blood and Tumor tissue	Female patients with hormone-receptor positive (HR$^+$) breast cancer were recruited from the Churchil Hospital, Oxford, United Kingdom. Ethical approval was given by the NHS South Central - Oxford C Research Ethics Committee (REC no. 19/SC/0173) under the Oxford Radcliffe Biobank (ORB) reference number 21/A053.	N/A
**Chemicals, peptides, and recombinant proteins**		
ACTL8 overlapping peptide pool	JPT	Cat#PM-ACTL8
Her2 overlapping peptide pool	JPT	Cat#PM-ERB_ICD; PM-ERB_ECD
MAGE-A1 overlapping peptide pool	Sigma-Aldrich	N/A
MAGE-A3 overlapping peptide pool	Sigma-Aldrich	N/A
NY-ESO-1 overlapping peptide pool	Sigma-Aldrich	N/A
PLAC1 overlapping peptide pool	JPT	Cat#PM-PLAC1
PRAME overlapping peptide pool	JPT	Cat#PM-OIP4
SALL4 overlapping peptide pool	Sigma-Aldrich	N/A
SOX2 overlapping peptide pool	Sigma-Aldrich	N/A
SSX2 overlapping peptide pool	Sigma-Aldrich	N/A
**Critical commercial assays**		
SMARTer Human TCR a/b Profiling Kit v2	Takara	Cat#634779
**Deposited data**		
TRON cell line portal (TCLP)	Scholtalbers et al.[19]	N/A
TCGA dataset	RTCGA package in R	N/A
scRNA-seq dataset 1	Wu et al.[24]	GEO: GSE176078
scRNA-seq dataset 2	Tietscher et al.[23]	EMBL-EBI under accession number ArrayExpress: E-MTAB-10607
VDJ database	Shugay et al.[62]	N/A

(*Continued on next page*)

Continued		
REAGENT or RESOURCE	SOURCE	IDENTIFIER
Experimental models: Cell lines		
Human: BT20 breast tumor cell line	Dr. Francesca Buffa	RRID:CVCL_0178
Human: HCC1937 breast tumor cell line	Dr. Francesca Buffa	RRID:CVCL_0290
Human: MCF-7 breast tumor cell line	Dr. Francesca Buffa	RRID:CVCL_0031
Human: MDA-MB-231 breast tumor cell line	Dr. Francesca Buffa	RRID:CVCL_0062
Human: SUM159PT breast tumor cell line	Dr. Francesca Buffa	RRID:CVCL_5423
Human: MDA-MB-436 breast tumor cell line	Dr. Francesca Buffa	RRID:CVCL_0623
Human: Tumor reactive CD8 T cell lines	This paper	N/A
Software and algorithms		
GraphPad Prism v10	Dotmatics	N/A
FlowJo v10	BD Biosciences	N/A
MiXCR v.3.0.13	Bolotin et al.[63]	N/A
immunarch v. 0.9.1	ImmunoMind Team[64]	N/A
circlize v.0.4.16	Gu et al.[65]	N/A
turboGliph v. 0.99.2	Hetzel et al.[66]	N/A
ggseqlogo v. 0.2	Wagih et al.[67]	N/A
ggplot2 v.3*.5.1*	Shugay et al.[68]	N/A
Seurat v5.0.1	Hao et al.[69]	N/A
SAVER v1.1.3	Huang et al.[70]	N/A

EXPERIMENTAL MODEL AND STUDY PARTICIPANT DETAILS

Human participants

Female patients with hormone-receptor positive (HR^+) breast cancer (Table 1) were recruited from the Churchil Hospital, Oxford, United Kingdom, between August 2021 and November 2023. All patients provided voluntary written informed consent. Ethical approval was given by the NHS South Central - Oxford C Research Ethics Committee (REC no. 19/SC/0173) under the Oxford Radcliffe Biobank (ORB) reference number 21/A053. On the day the patients were receiving surgery, blood was collected before the procedure. After the surgery, a 0.5 cm punch biopsy of the tumor was collected together with up to 5 mg of paratumor tissue with no visible sign of malignancy, and stored at 4°C in RPMI-1640 (Thermo Fisher Scientific) for a maximum of 20 h. Only patients with sufficient material collected were included in the study.

Breast cancer cell lines

The established breast cancer cell lines BT20 (RRID:CVCL_0178), HCC1937 (RRID:CVCL_0290), MCF-7 (RRID:CVCL_0031), MDA-MB-231 (RRID:CVCL_0062), SUM159PT (RRID:CVCL_5423) and MDA-MB-436 (RRID:CVCL_0623) were kindly provided by Dr. Francesca Buffa. The cells were cultured in DMEM medium (Thermo Fisher Scientific) supplemented with 10% fetal bovine serum (FBS; Sigma-Aldrich) and 1% antibiotic-antimycotic (Sigma-Aldrich). All cell lines were free of mycoplasma contamination. The HLA typing of the cell lines were confirmed by sequencing exon 2 and 3 of each locus (Table S2).

METHOD DETAILS

Tissue processing and lysate preparation

Single cell suspensions of the tumor and paratumor tissues were obtained by cutting the tissue into small pieces and performing enzymatic dissociation using the human tumor dissociation kit (Miltenyi Biotech), following the supplier's protocol. Cells were centrifuged and filtered through a 100-μm strainer to remove undigested fragments. The protein lysate was prepared as previously reported,[9] using the undigested fragments together with 20% of the single-cell suspension. Briefly, the cells and fragments were resuspended in PBS and submitting to 5 freeze-thaw cycles, after which they were centrifuged, and the supernatant used as tumor lysate. Protein quantification was done using the Pierce Bradford Plus Protein Assay Kit (Thermo Fisher Scientific), following the supplier's instructions.

Blood processing, T cell isolation and monocyte-derived dendritic cells differentiation

Peripheral blood mononuclear cells (PBMCs) were obtained using Lymphoprep (STEMCELL Technologies) density gradient isolation. Monocytes were positively selected using CD14 MicroBeads (Miltenyi Biotec), following the supplier's protocol.

Monocyte-derived dendritic cells were generated, as previously described,[71] by culturing the purified monocytes in AIM-V medium (Thermo Fisher Scientific) supplemented with 50ng/mL IL-4 (Peprotech) and 50ng/mL GM-CSF (Peprotech) for 7 days 50ng/mL TNF-α (Peprotech) was added over the last 2 days to activate the cells. T cells were isolated from the monocyte-depleted PBMCs by negative selection using Pan T cell Isolation Kit (Miltenyi Biotec), following the supplier's instructions.

Proliferation assay and isolation of lysate-specific T cells

The proliferation assay was performed as previously reported.[9,72] T cells were labeled with 2.5 μM carboxyfluorescein succinimidyl ester (CFSE; Thermo Fisher Scientific), while dendritic cells were labeled with 10 μM Cell Trace Violet (CTV; Thermo Fisher Scientific). Dendritic cells (DC) were loaded with tissue lysates at 10 μg of protein/mL for half an hour under rotation. The cells were then seeded in wells of a 96-well U-bottom culture plate at a 10:1 lymphocyte:DC ratio in complete medium (CM) consisting of RPMI-1640 supplemented with 5% human AB serum (Sigma-Aldrich), 1% antibiotic-antimycotic (Sigma-Aldrich), 1 mM sodium pyruvate (Thermo Fisher Scientific), 1% Non-essential Amino Acid mixture 100x (Thermo Fisher Scientific), 2 mM GlutaMAX supplement (Thermo Fisher Scientific) and 5×10^{-5} M β-Mercaptoethanol (Thermo Fisher Scientific). CD3/CD28 DynaBeads (Thermo Fisher Scientific) were used as a positive control for activation. After three to five days, Proleukin (recombinant IL-2) at a final 50 U/mL was added to the culture. After three more days, the cells were harvested and stained with Live/Dead aqua (Thermo Fisher Scientific) and antibodies specific for CD8, CD4, CD25 and ICOS. Proliferating CD8 T cells ($CD8^+CFSE^{low}CD25^+$) were sorted in bulk or as single cells, using a BD FACS Aria III (BD Biosciences). The relative proliferation was calculated by subtracting the frequency of $CFSE^{low}CD25^+$ on CD8 T cells stimulated with unloaded DC from the frequency of $CFSE^{low}CD25^+$ on CD8 T cells stimulated with tissue-loaded DC, and scaling negative values to zero. A patient was considered to have a detectable circulating tumor-reactive CD8 T cell response if the percentage of $CFSE^{low}CD25^+$ CD8 T cells after coculture with DCs loaded with tumor lysate was higher than the baseline proliferation with unloaded DCs.

Generation of tumor-reactive T cell lines and T cell clones

To generate T cell clones, a single cell was sorted in each well of a 96 U-bottom plate, while the T cell lines were generated by bulk sorting all cells of interest in the same tube. The T cell lines and clones were established and maintained as previously described, by expanding the cells for 10–14 days using irradiated allogeneic PBMCs and phytohemagglutinin (PHA).[72,73] The cells were kept in complete medium supplemented with 200 U/mL IL-2 (Proleukin), 1ng/mL IL-7 (Peprotech) and 1ng/mL IL-15 (Peprotech).

Evaluation of T cell response against tumor cell lines and CTA peptide pools

Intracellular cytokine staining (ICS) was performed as described previously described.[74] T cell clones were cocultured with tumor cell lines in R10 medium, composed of RPMI-1640 (Thermo Fisher Scientific) supplemented with 10% fetal bovine serum (FBS; Sigma-Aldrich) and 1% antibiotic-antimycotic (Sigma-Aldrich), in the presence of CD107a antibody and 3 μg/mL brefeldin A (Thermo Fisher Scientific). MHC blocking was achieved by culturing the tumor cell lines with 40 μg/mL of HLA-ABC (Biolegend, clone W6/32), HLA-A2 (Sigma, clone BB7.2) or isotype control (Biolegend) for 60 min, before adding the T cells. To evaluate the response against CTAs, tumor-reactive T cell lines were cultured in CM in the presence of overlapping peptide pools for ACTL8 (JPT), HER2 (JPT), MAGE-A1 (Sigma-Aldrich), MAGE-A3 (Sigma-Aldrich), NY-ESO-1 (Sigma-Aldrich), PLAC1 (JPT), PRAME (JPT), SALL4 (Sigma-Aldrich), SOX2 (Sigma-Aldrich) and SSX2 (Sigma-Aldrich).

Cells were cultured for 4 h, and then harvested, stained with Live/Dead acqua (Thermo Fisher Scientific), fixed with Fixation buffer (Biolegend), and stained with antibodies against CD8, CD4, IFN-γ, TNF-α and MIP1β in Permeabilization buffer (Thermo Fisher Scientific). Cells were then acquired on a LSRFortessa X-20 (BD Bioscience) and data analyzed on FlowJo v.10.9 (BD Biosciences).

Killing assay

Tumor cell lines were labeled with Far Red dye (Thermo Fisher Scientific) and co-cultured with tumor-reactive T cell clones in a flat bottom 96-well plate at different effector:target (E:T) ratio in technical triplicates in R10 medium. 0.1 μL CellTox (Promega) was added into each well and the plate was placed into the IncuCyte S3 imaging system (Sartorius) to track the green fluorescent signal accumulation for each well at every two to three-hour intervals. The Relative Green Intensity was calculated by using the total integrated intensity (TII) on the green channel, using the following formula for each timepoint: (TII of the coculture) – (TII of T cells only) – (TII of tumor cells only). 48 h after coculture, the cells from the technical replicates were harvested, pooled, stained with Zombie Red Dye (Biolegend), and CTV (Thermo Fisher Scientific) labeled tumor cells were then added as references cells for FACS analysis. Cells were acquired on a LSRFortessa X-20 (BD Bioscience) and data analyzed on FlowJo v.10.9 (BD Biosciences). The percentage of T cell mediated killing for each well was calculated with the following equation: % cytotoxicity = 100 − ((% target cell/% reference cell)/(% target cell control/% reference cell) × 100). The % target cell control was determined by culturing tumor cells in the absence of tumor-reactive T cells.

Generation of CD8 TIL cell lines for TCR sequencing

To generate the tumor-infiltrating CD8 T cell lines used for TCR sequencing, 20% of the total tumor single-cell suspension obtained after tissue dissociation was cultured overnight in R10 medium, and the non-adherent cells were recovered and expanded for 14 days using irradiated allogeneic PBMCs and phytohemagglutinin (PHA), as previously described.[72,73] On day 14 post-expansion,

CellPress
OPEN ACCESS

at least 15% of the T cell line was stained with Live/Dead aqua (Thermo Fisher Scientific) and antibodies specific for CD3, CD4 and CD8. The CD8 T cells were then sorted using a BD FACS Aria III (BD Biosciences). If less than $2x10^5$ CD8 TILs were sorted, the cells were submitted to another round of expansion before having their TCR sequenced.

Deep sequencing of the TCR of T cell lines and clones

At least 15% of cells from each expanded T cell line, and at least $1x10^5$ cells from each clone were harvested at day 14 post-expansion for TCR sequencing. Given the substantial expansion induced by the feeders during the generation of the T cell lines, the diversity of TCR sequences should be represented in 15% of the T cell lines. For the tumor-reactive T cell lines, we harvested between $5x10^5$ and $1x10^6$ CD8 T cells for the TCR sequencing. Using the Poisson distribution, the probability of not sampling a clone that has a frequency of 0.01% in the population, when taking 15% of the material and having at least $5x10^5$ cells is extremely small (1.928×10^{-22}), ensuring that the sampling strategy retains the TCR repertoire diversity.

The cells were washed with phosphate-buffered saline (PBS), and had their RNA extracted using the RNeasy Plus Mini or Micro Kit (QIAGEN) and quantified using NanoDrop (Thermo Fisher Scientific). The RNA of the lines and clones was then used to generate full-length TCR repertoire libraries for Illumina Sequencing using the SMARTer Human TCR a/b Profiling Kit v2 (Takara), following the supplier's instructions, and as previously described.[74] Briefly, 100-1000ng RNA, depending on sample availability, was used as input for cDNA conversion using primers containing unique molecular identifiers (UMIs) to facilitate PCR error correction. The cDNA sequences of the variable regions of TCR-α and TCR-β transcripts were then amplified in two rounds of PCR using nested PCR primers. The second PCR reaction also utilized primers containing unique dual indexes (UDIs), allowing for sample barcoding. PCR products were then purified using SPRIselect beads (Beckman Coulter) and eluted in EB buffer (Qiagen). The quantity and quality of cDNA libraries were checked on a Tapestation 4200 system using the D5000 reagents (Agilent). Sequencing was performed using MiSeq reagent Kit v.3 (600 cycles) on MiSeq (Illumina) with MiSeq Control Software v.2.6.2.1.

Immunohistochemistry analysis

Formalin fixed Paraffin Embedded (FFPE) breast tumor resections were cut at 2 μm thickness and a heat-induced epitope retrieval protocol was performed using the Bond ER2 solution (Leica). Adjacent slides were separately stained against CD8 (Leica; clone 4B11) and CD3 (Leica, clone LN10). The detection system used was Bond refine DS9800 (Leica). Image analysis was done using the software QuPath (version 0.5.1).[75] The percentage of area with CD8 and CD3 staining was determined on the full tumor area present on the slides. Alternatively, the number of CD8 T cells was calculated in a maximum of 10 random regions of interest (ROIs) of 1 mm^2 each inside the tumor of 20 patients. The number of CD8 T cells was also determined in 10 paratumor ROI. TLS identification and quantification was done by a pathologist that identified structured areas with the presence of a mixed T cell and plasma cell population.

Tumor-reactive TCR repertoire analysis

BCL files were demultiplexed and converted to FASTQ format using bcl2fastq v.2.20.0.422 (Illumina). TCR clones were extracted using MiXCR v.3.0.13,[63] and the resulting output files (TRA and TRB) were parsed into R using the repLoad function of immunarch v. 0.9.1.[64] The files were filtered to contain only unique clonotypes (defined as V and J gene usage and CDR3β sequence) that had a count of at least 10. V-gene usage was plotted using the geneUsage function of immunarch. Circos plots showing V-J usage of the TRB of each unique clonotype were created using circlize v.0.4.16.[65] For repertoires with more than 200 unique clonotypes, only the sequences occupying more than 0.05% of the repertoire were drawn. The heatmap of CDR3 repertoire overlap was drawn using the repOverlap function of immunarch. The GLIPH2 similarity analysis was done using turboGliph v. 0.99.2.[66] The sequence logo plot was done using ggseqlogo v. 0.2.[67]

Determination of frequency of tumor-reactive and pathogen-specific TRB in tumor

To determine the frequency of tumor-reactive TIL, two different approaches were used. First, the number of CDR3β amino acid sequences from the circulating tumor-reactive CD8 T cells that were identical to the CDR3β amino acid sequences from the CD8 T cells infiltrating the tumor were divided by the total number of clonotypes in the tumor. A second approach includes as tumor-reactive the CDR3β sequences that were similar by GLIPH2 analysis to circulating tumor-reactive CDR3β. To determine the frequency of pathogen-reactive TRB, the CDR3β amino acid sequences from the tumor were compared to CDR3β sequences from the VDJdb.[62] A TRB was considered as being pathogen-specific if the CDR3β amino acid sequence completely matched a VDJdb entry described to be specific for a pathogen and restricted to an HLA allele that matched the patient's HLA.

Analysis of expression of tumor antigens on the breast cancer cell lines

To analyze the expression of tumor antigens on breast cancer cell lines, the mutation data and the antigen expression data of TRON cell line portal (TCLP) was used.[19] 218 genes described as a CTA on the CTdatabase[76] were present in the TRON dataset. A cell line was defined as expressing an antigen if their gene expression was greater or equal than 5. When comparing individual lines, a Venn diagram was drawn with the number of CTA and neoantigen expressed and shared by each cell line (Figures S2D and S2E). When comparing all cell lines with the same subtype (Figure S2A), a CTA was considered shared if it was expressed by at least one of the lines in each subtype.

Analysis of expression of tumor antigens on cancer patients

The TCGA dataset[18] was used to determine the neoantigen count and expression of CTAs, and selected tumor antigens on breast cancer patients with Luminal A, Luminal B and Normal molecular intrinsic tumor subtypes, which are subtypes enriched for hormone receptor expression.[77] The normalized read counts of selected tumor antigens were plotted using ggplot2 v.3*.5.1.*[68] For the calculation of the number of expressed CTAs per patient, a gene expression cut off of 5 was used.

Single-cell RNA sequencing data acquisition and analysis

Previously published scRNA-seq datasets were obtained from the Gene Expression Omnibus (GEO) (accession number GEO: GSE176078)[24] and the ArrayExpress database at EMBL-EBI (accession number ArrayExpress: E-MTAB-10607).[23] Gene expression matrices and available cell and patient metadata were imported into R (v4.3.2) and analyzed using the Seurat (v5.0.1)[69] package. All parameters were left as the default value unless specified. Quality control filtering was performed to exclude cells with more than 5,000 detected genes. Both datasets had already excluded cells with greater than 20% mitochondrial gene expression. Each dataset was analyzed individually. Data normalization was performed using SCTransform using 3,000 variable features, and sample integration was performed using Harmony (Seurat v5.0.1). Dimensional reduction was performed by principal component analysis (PCA) and uniform manifold approximation and projection (UMAP). Cell type annotations were transferred from original publication. CD8 T cells were selected by first identifying T/NK cells, as annotated by both datasets, followed by selecting CD8 and mixed clusters. CD8A expression was inputted using SAVER (v1.1.3),[70] and positive cells were manually selected based on the bimodal distribution. Lastly, to ensure a highly pure CD8 population, events expressing CD4 > 0.5 and CD8 = 0 were excluded. Clustering of dataset 2 was done using 10 dimensions, and a resolution of 0.1 was used for the first clustering to remove the main NK and CD4 cluster, followed by clustering the purified CD8 T cells at resolution 0.7. For visualization and calculation of module scores, raw counts were normalized with the NormalizeData function with method = "LogNormalize" (Seurat v5.0.1). Module score was calculated with the AddModuleScore function (Seurat v5.0.1).[26]

QUANTIFICATION AND STATISTICAL ANALYSIS

Statistical analysis

Statistical analyses were conducted using Prism v. 10 (GraphPad). The number of patients and biological and technical repeats can be found in the figure legends. Correlation analyses were performed using non-parametric Spearman rank correlation. Statistically significant differences between two normally distributed variables were assessed using two-tailed paired or unpaired t-test. Non-parametric Mann-Whitney test was used for unpaired variables, and Wilcoxon matched-pairs signed rank test were used for paired samples, when the variables that did not pass the normality test. One-way ANOVA with Tukey's multiple-comparison test or two-way ANOVA with Tukey's multiple-comparison test was performed to compare two or more groups. Statistical significance was set as $^*p < 0.05$, $^{**}p < 0.01$ and $^{***}p < 0.001$.

Cell Reports Medicine, Volume *6*

Supplemental information

Tumor-specific CD8 T cell characterization in HR+ breast cancer reveals an impaired antitumoral response in patients with lymph node metastasis

Mariana Pereira Pinho, Elie Antoun, Balraj Sandhar, Ting Shu, Fei Gao, Xiaobao Yang, Adam Bates, Lucia Cerundolo, Megat H.B.A. Hamid, David Maldonado-Perez, Renuka Teague, Eve Warner, Lucinda Winter, Nasullah Khalid Alham, Clare Verrill, Simon R. Lord, Timothy Rostron, Sally-Ann Clark, Craig Waugh, Paul Sopp, Chris Conlon, Ricardo A. Fernandes, Adrian L. Harris, Yanchun Peng, Asha Adwani, and Tao Dong

Table S1: Correlation between the presence of a detectable tumor-reactive CD8 T cell response and clinical characteristics of the patients. The p-value was calculated using Fisher's exact test for the contingency table (Preoperative treatment) and t-test and Mann-Whitney for normally distributed variables, or not, respectively. A patient was considered to have a detectable circulating tumor-reactive CD8 T cell response if the percentage of $CFSE^{low}CD25^{+}$ CD8 T cells after coculture with DCs loaded with tumor lysate was higher than the baseline proliferation with unloaded DCs. Related to Figure 1.

Characteristic	Patients with an undetectable circulating tumor-reactive T cell response (n = 7)	Patients with a detectable circulating tumor-reactive T cell response (n = 16)	p-value
Age - median (range)	65 (51-83)	69 (39-86)	0.6251
Tumor size (mm) - median (range)	27 (17.5-60)	36 (19-120)	0.3155
Preop treatment (Letrozole):			
Yes - n (%)	1 (14.3)	2 (12.5)	>0.999
No - n (%)	6 (85.7)	14 (87.5)	
Progesterone score - median (range)	2 (0-8)	6 (0-8)	0.2703
Tumor grade - median (range)	2 (1-3)	2.5 (2-3)	0.2597

Table S2: Breast cancer subtype and HLA typing of the established cancer cell lines. TNBC: Triple-negative breast cancer. HR^{+}: Hormone receptor positive. Related to STAR Methods and Figure 2.

Cell line	Subtype	HLA-A		HLA-B		HLA-C	
BT20	TNBC	24:02	24:03	15:01	38:01	03:03	12:03
HCC1937	TNBC	23:01	24:02	07:02	40:01	03:04	07:02
MCF-7	HR^{+}	02:01	02:01	18:01	44:02	05:01/03	05:01/03
MDA-MB-231	TNBC	02:01	02:17	40:02	41:01	02:02	17:01/02
SUM159PT	TNBC	02:01	24:02	15:01	51:01	03:03	15:02
MDA-MB-436	TNBC	01:01/04N	01:01/04N	08:01	08:01	07:01	07:01

Table S3: HLA typing of breast cancer patients. Related to Figure 2.

Patient ID	HLA-A		HLA-B		HLA-C	
Br1	02:01	24:02	07:02	55:01	03:03	07:02
Br4	01:01/04N	03:01	53:01:00	57:01:00	04:01	06:02
Br6	32:01:00	32:01:00	08:01	14:01	07:01	08:02
Br7	01:01/04N	29:02	08:01	44:03	07:01	16:01
Br8	02:01	29:02:00	44:02:00	44:03:00	05:01/03	16:01
Br10	02:01	23:01	40:02:00	44:03:00	02:02	04:01
Br14	01:01/04N	02:01	07:02	15:01	03:04	07:02
Br15	01:01/04N	29:02	08:01	44:03	07:01	16:01
Br16	01:01/04N	02:01	08:01	44:02	05:01/03	07:01
Br17	02:01	02:01	15:01	51:01	03:03	15:02
Br18	24:02:00	24:02:00	14:02	35:03	04:01	08:02
Br19	02:01	26:01	38:01	44:02	05:01/03	12:03
Br20	01:01/04N	33:05	14:02	57:01	06:02	08:02
Br21	02:01	31:01	15:01	40:01	03:04	03:04
Br22	02:01	02:01	15:01	51:01	02:02	03:04
Br23	01:01/04N	02:01	08:01	15:01	03:03	07:01
Br26	02:01	03:01	07:02	14:01	07:02	08:02
Br27	01:01/04N	24:02	08:01	52:01	07:01	12:02
Br29	02:01	68:01	08:01	51:01	06:02	07:01
Br30	01:01/04N	02:01	40:01	57:01	03:04	06:02
Br31	02:01	02:01	37:01	44:02	05:01/03	06:02
Br32	01:01/04N	02:01	08:01	44:02	05:01/03	07:01
Br37	01:01/04N	02:01	08:01	44:02:00	07:01	12:03
Br24	02:01	02:01	08:01	44:02:00	05:01/03	07:01
Br25	02:01	02:05	07:02	49:01:00	07:01	07:02

Table S4: CDR3α and CDR3β amino acid sequences of the TCRs from the T cell clones isolated from patient Br1 and Br23. Related to STAR Methods and Figure 2.

T cell clone	CDR3α	CDR3β
Br1 clone 1	CLVGEDKLVF	CATHQGAGEQYF
Br1 clone 2	CAASGSNTGNQFYF	CASSSQAGIQTDTQYF
Br23 clone 3	CLVGSNDYKLSF (97%)<br>CAVPRKGRPSNTGKLIF (3%)	CASSQVARDTDTYYGYTF
Br23 clone 4	CALDSNYQLIW	CSGPSLPGGYTF

Table S5: CDR3 amino acid sequences of the TCR alpha and beta chains that are shared between tumor-reactive T cell lines. Related to Figure 3.

Patients	TCR chain	CDR3 sequence
Br6, Br8	alpha	CAALNYGGSQGNLIF
Br8, Br37	alpha	CAASSGGYQKVTF
Br7, Br8	alpha	CALNDYKLSF
Br26, Br27	alpha	CAMREGQMGDDKIIF
Br8, Br23	alpha	CAMREGYNDMRF
Br7, Br8	alpha	CAVNTGGFKTIF
Br7, Br8	alpha	CAVNTNAGKSTF
Br16, Br37	alpha	CAVPNDYKLSF
Br6, Br10	alpha	CAVRGDYKLSF
Br6, Br8	alpha	CVVSEGSGTYKYIF
Br6, Br8	beta	CASSLAGGAYEQYF
Br8,Br10	beta	CASSLGGSSTDTQYF
Br6, Br37	beta	CASSLGRNTEAFF
Br8, Br37	beta	CASSRFTDTQYF

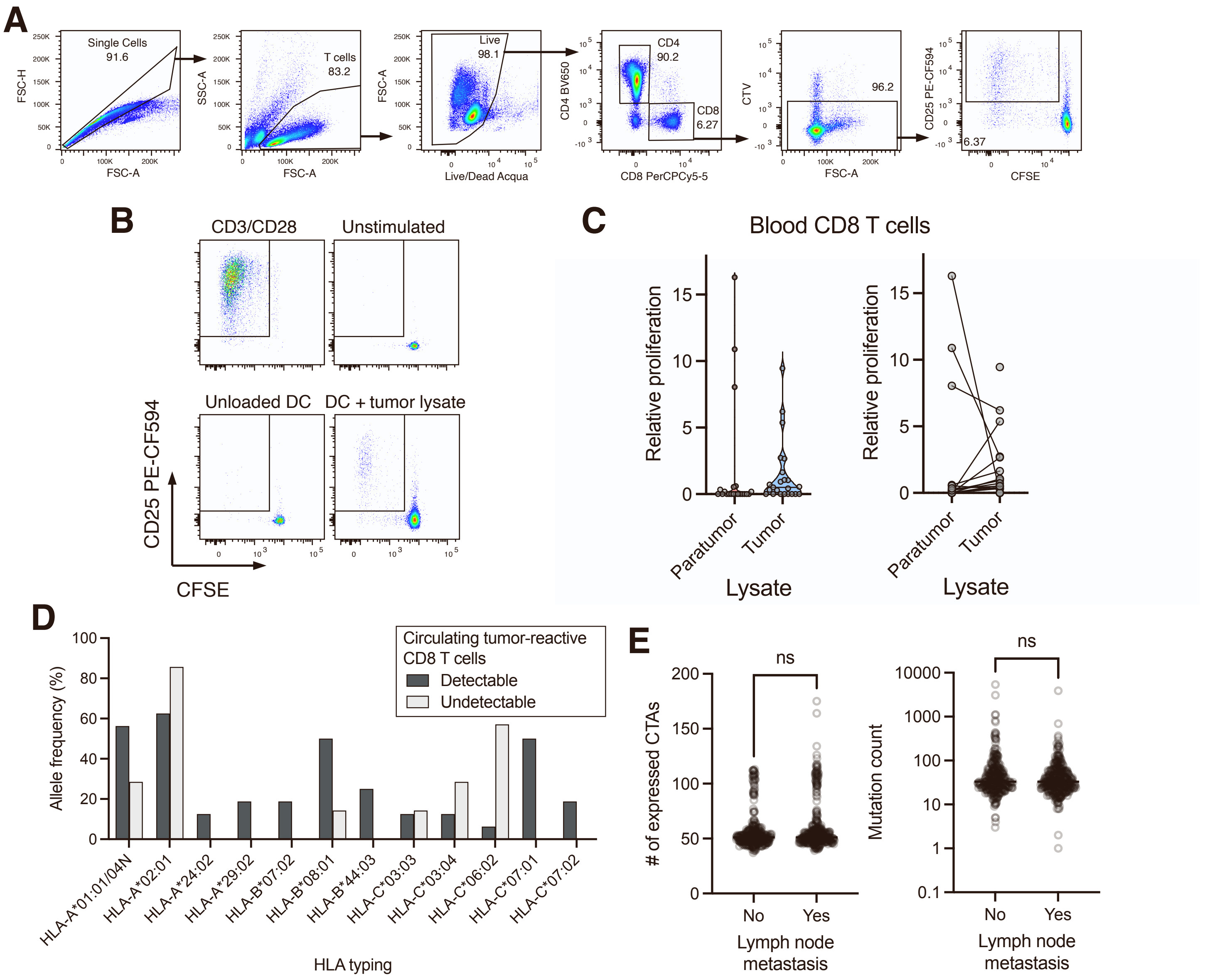

Figure S1: Detection of blood tumor-reactive CD8 T cell responses. (A) Gating strategy of the T cell proliferation assay. (B) Representative dot plots of CFSE and CD25 expression after proliferation assay. (C) Graph showing the normalized percentage of CFSElowCD25+ CD8 T cells after culture with DC loaded with tumor lysate (n = 23) or paratumor lysate (n = 21). (D) Graph showing HLA class I allele frequency, which corresponds to the percentage of patients within either the detectable (n = 16; dark bars) or undetectable (n = 7; light bars) group that have a specific HLA allele. (E) Number of expressed CTAs (p=0.0629) and mutation count (p=0.1726) in TCGA patients with (n=383) and without (n=334) lymph node metastasis. Mann-Whitney test. Related to Figure 1.

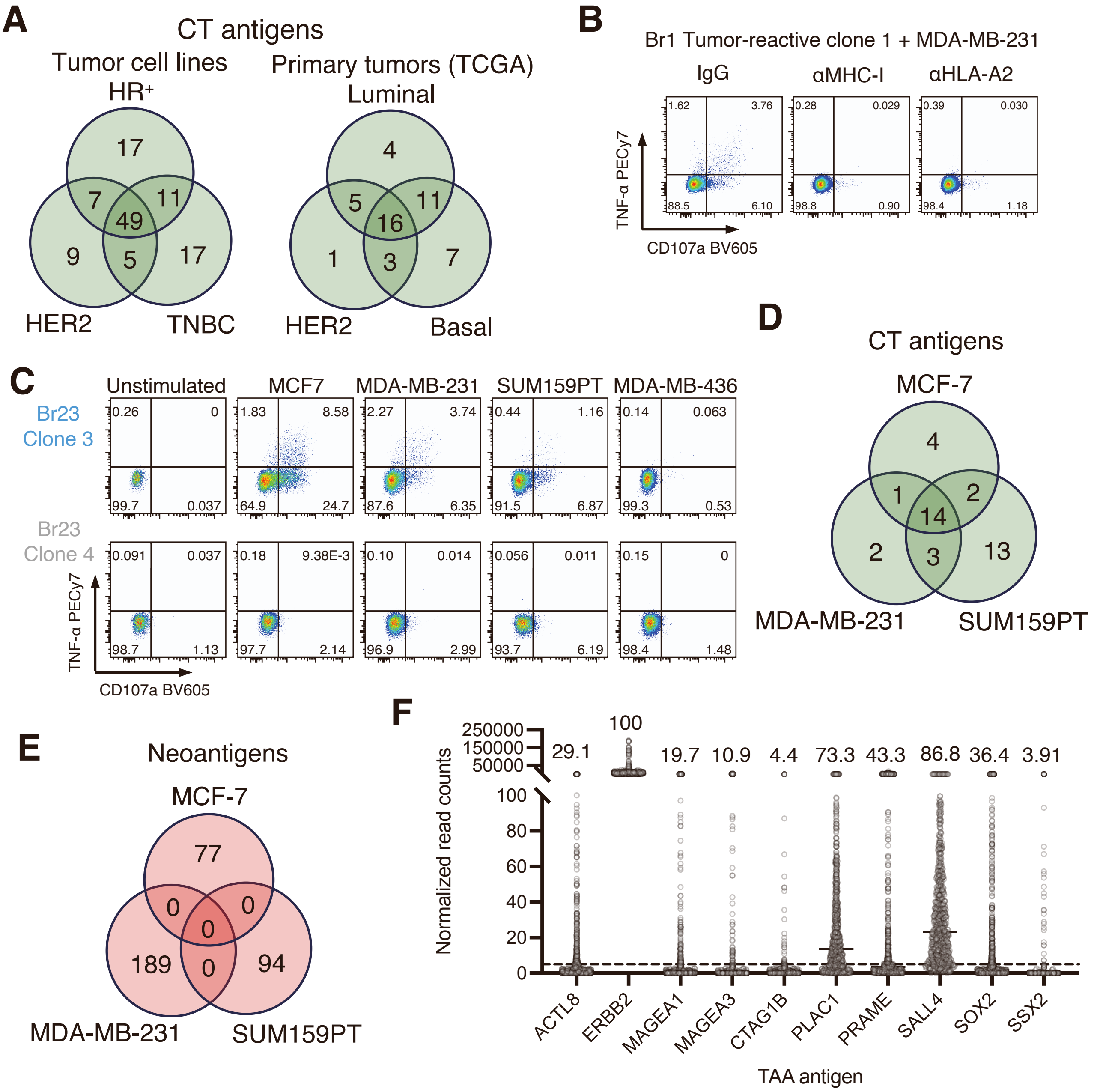

Figure S2: Expression of tumor antigens and specificity of tumor-reactive CD8 T cell clones. (A) Venn diagram showing the number of CTA expressed and shared between cell lines (left) or primary tumors (right) of different subtypes, using the TRON and TCGA dataset, respectively. Number of primary tumors analysed: Luminal n=696; HER2 n=78; Basal n=171. Number of cell lines analysed: HR+ n=9; HER2 n=9; TNBC n=17 (B) Dot plots showing CD107a and TNF-α expression on Br1 tumor-reactive T cell clone 1 cocultured with MDA-MB-231 in the presence of antibodies to block MHC-I, HLA-A2 or an isotype control antibody. (C) Representative graph showing CD107a and TNF- expression on Br23 tumor-reactive T cell clones cultured in the presence of different breast cancer cell lines. (D) Venn diagram showing the number of CTA expressed and shared by each of three breast cancer cell lines (MCF-7, MDA-MB-231, SUM159PT), as determined using the TRON cell line portal dataset. (E) Venn diagram showing the number neoantigens expressed and shared by each of the three breast cancer cell lines, as determined using the TRON cell line portal dataset. (F) Normalized RNA expression of 10 tumor-associated antigens (TAA) in breast cancer patients of Luminal A, Luminal B and Normal molecular intrinsic tumor subtypes from the TCGA dataset (n=818). Related to Figure 2.

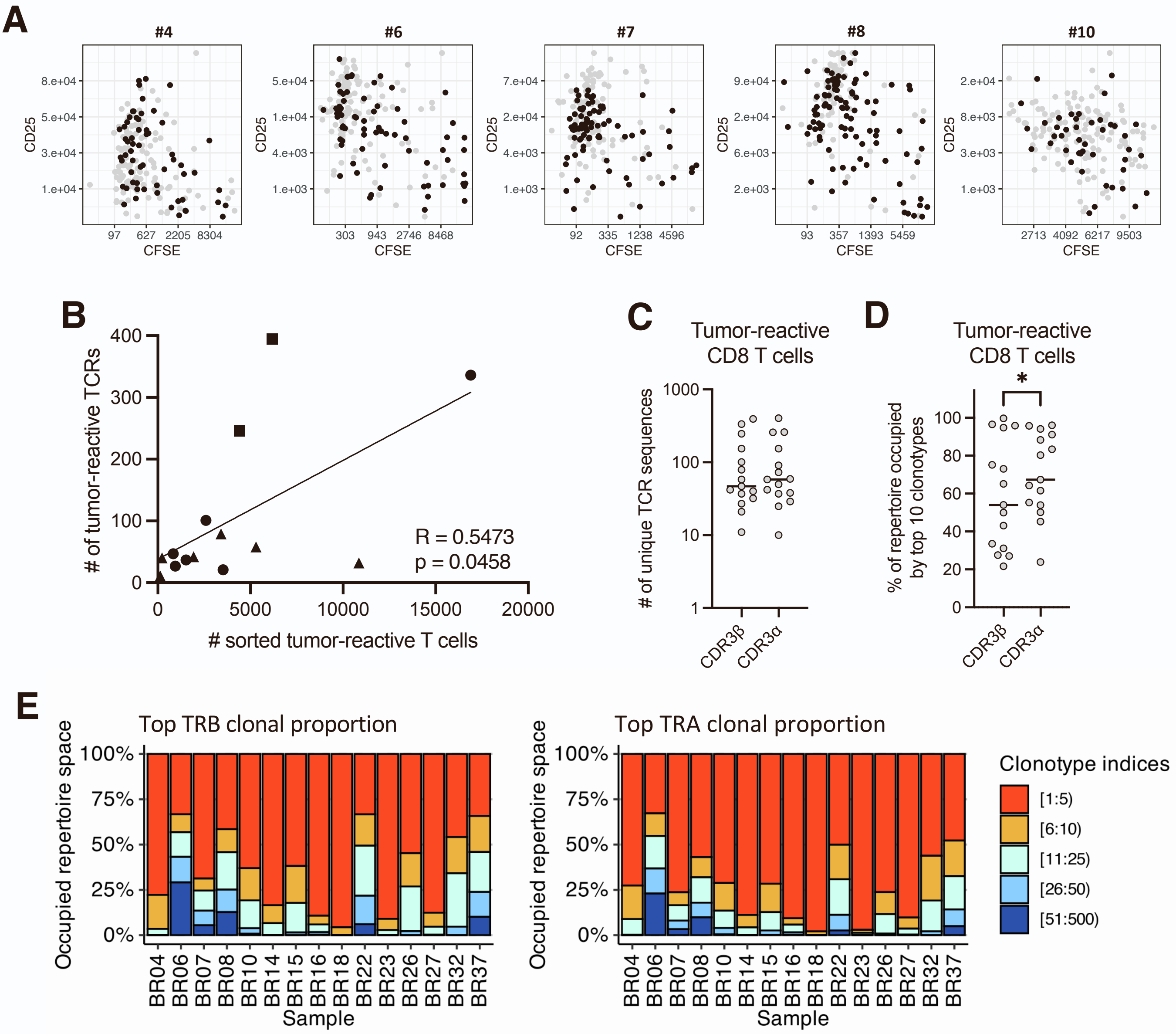

Figure S3: Analysis of tumor-reactive CD8 TCRs. (A) Dot plot showing the CFSE dilution and CD25 expression of each sorted individual CD8 T cell that was proliferating in the presence of the tumor lysate. Each single cell was expanded with feeders, and those that were successfully expanded into T cell clones are marked in black. (B) Correlation analysis between the number of tumor-reactive CDR3β and the number of sorted tumor-reactive T cells. Correlation analysis was performed using non-parametric Spearman rank correlation (p=0.0458). The shape of the dots represents the breast cancer subtype (circle = ductal; triangle = lobular; square = mucinous). (C) Number of unique CDR3 alpha and beta sequences in the tumor-reactive blood CD8 T cell lines. Each dot represents a different patient (n = 15; p=0.6001; Wilcoxon matched-pairs signed rank test). (D) Percentage of the repertoire occupied by the top 10 CDR3 alpha or beta clonotypes on the tumor-reactive T cell lines (n = 15; p=0.0147; two-tailed paired t-test). (E) Horizontal bar graph showing the proportion of the repertoire space occupied by the 5 most abundant clonotypes (red), or by the clonotypes with the indicated indices, in the TRB (left) or TRA (right). Related to and Figure 3.

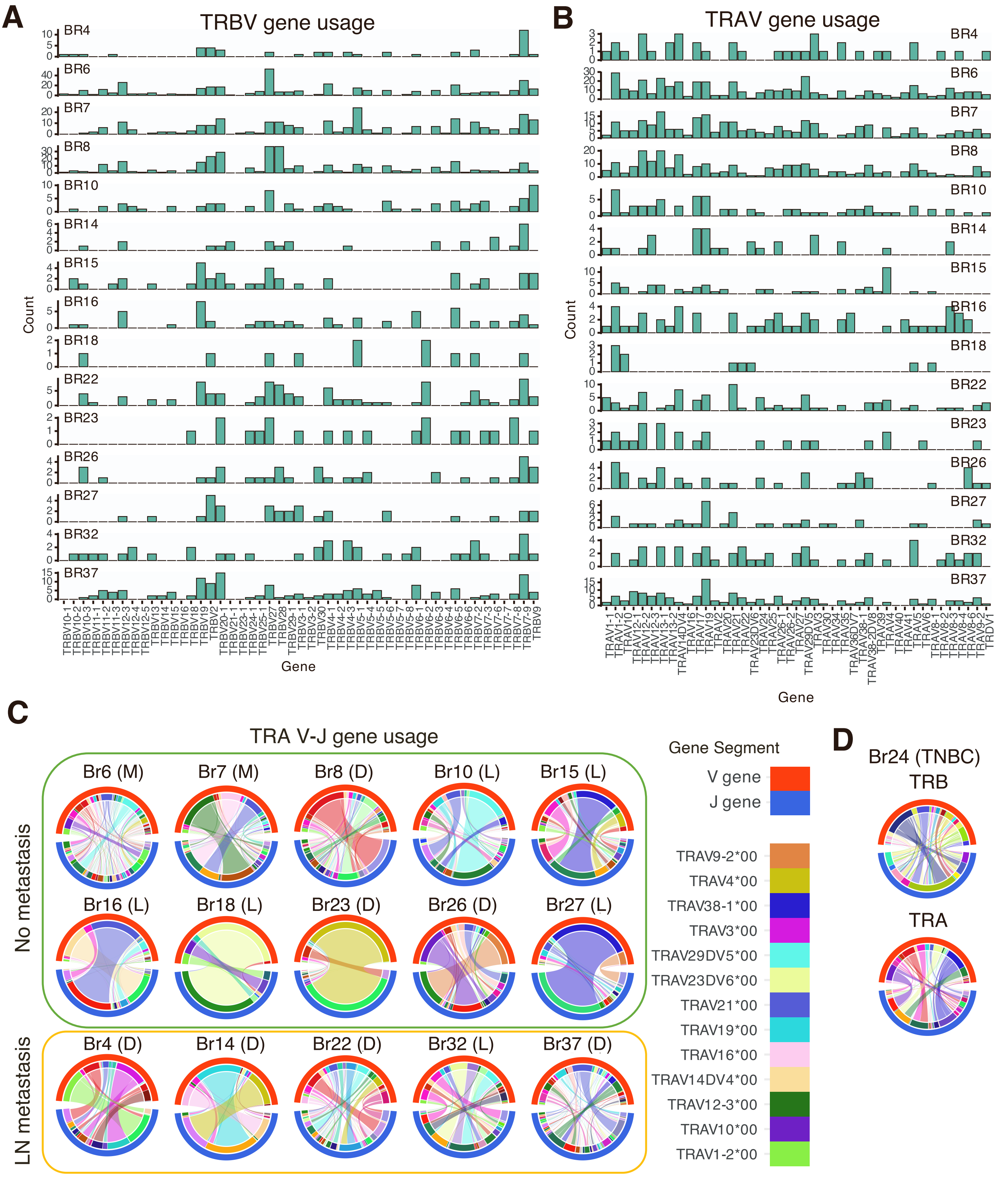

Figure S4: Tumor-reactive CD8 T cell repertoire diversity. (A) Plot showing the number of tumor-reactive clonotypes bearing each TRBV gene fragment in each patient. (B) Plot showing the number of tumor-reactive clonotypes bearing each TRAV gene fragment in each patient. (C)) V-J rearrangement circos plots of the T cell receptor alpha chain (TRA) in tumor-reactive T cell lines derived from each patient. Each plot represents the distribution of V (red outer arc) and J (blue outer arc) gene segment usage, with the connecting lines indicating specific V-J gene rearrangements for each TCR clonotype. The width of connecting lines reflects the relative abundance of that specific clonotype, with the color corresponding to the used TRAV segment. In parentheses next to each patient ID is the subtype of the tumor (L = lobular, D = ductal, M = mucinous). Samples are grouped based on the presence or absence of lymph node metastasis. (D) V-J rearrangement circos plot of the T cell receptor beta (TRB) alpha chain (TRA) of the tumor-reactive T cell line from a triple-negative breast cancer (TNBC) patient (Br24). Related to Figure 3.

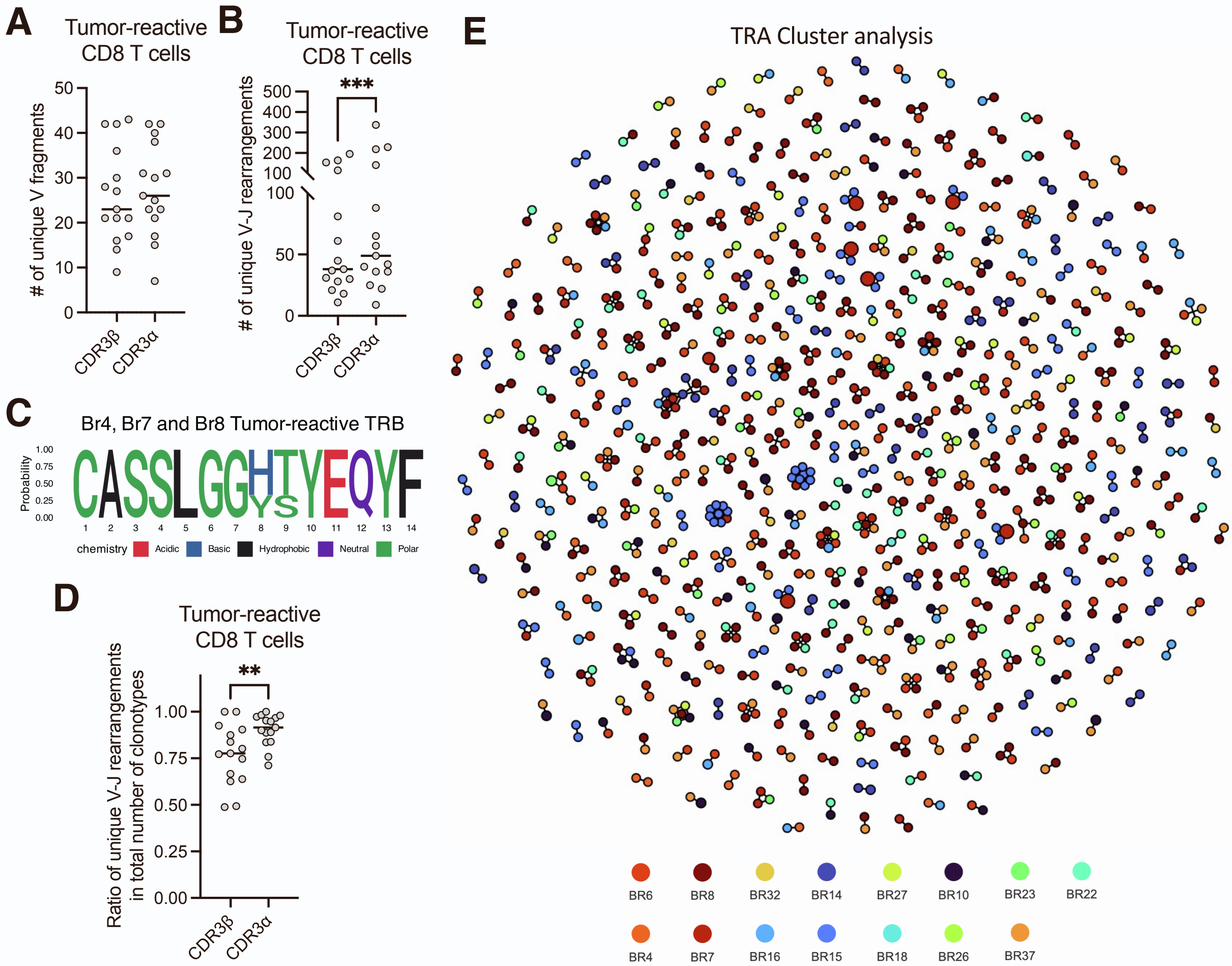

Figure S5: Diversity of TCR alpha and beta chain. (A) Number of unique V gene fragments used by the alpha and beta chain of tumor-reactive TCRs (n = 15; p=0.0777; two-tailed paired t-test). (B) Number of unique V-J rearrangements used by the alpha and beta chain of tumor-reactive TCRs (n = 15; p=0.0001; Wilcoxon matched-pairs signed rank test). (C) Sequence logo plot of a tumor-reactive CDR3β that is similar between three patients. (D) Ratio of unique V-J rearrangements used by the alpha and beta chain of tumor-reactive TCRs, in the total number of clonotypes (n = 15; p=0.0056; two-tailed paired t-test). (E) TRA cluster analysis of tumor-reactive CD8 T cells using the GLIPH2 algorithm. Each color represents a different patient. The link highlights TCRs that are similar based on global alignment of the CDR3α. Related to Figure 3.

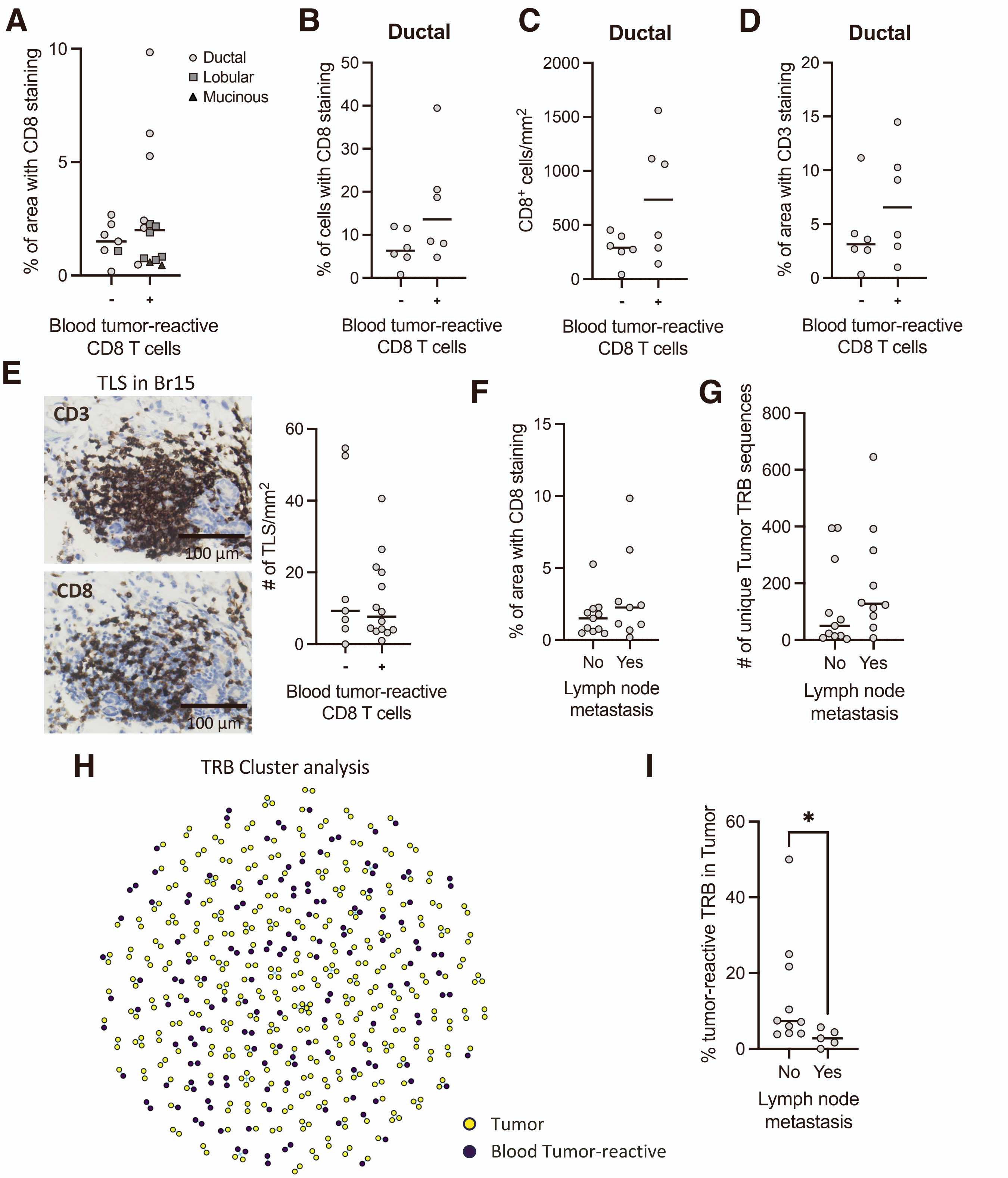

Figure S6: Analysis of tumor-reactive TILs. (A) Percentage of the total tumor area with CD8 staining in patients with (n=14) and without (n=7) a detected blood tumor-reactive T cell response (p=0.7433; Mann-Whitney test). (B) Percentage of $CD8^+$ cells in 10 randomly selected tumor areas in ductal breast cancer patients with (n=6) and without (n=6) a detected blood tumor-reactive T cell response (p=0.1085; two-tailed unpaired t-test). (C) Number of $CD8^+$ cells per mm^3 of tumor area, calculated using 10 randomly selected areas inside ductal breast tumors, in ductal patients with (n=6) and without (n=6) a detected blood tumor-reactive T cell response (p=0.0732; two-tailed unpaired t-test). (D) Graph showing the lack of difference in the percentage of the total tumor area with CD3 staining in ductal patients with (n=6) and without (n=6) a detected blood tumor-reactive T cell response (p=0.2904; two-tailed unpaired t-test). (E) Representative IHC of the CD3 and CD8 staining in a region characteristic of tertiary lymphoid structures (TLS), and graph showing the number of TLS present in patients with (n=14) and without (n=7) a detected blood tumor-reactive T cell response (p=0.5846; Mann-Whitney test). (F) Percentage of the total tumor area with CD8 staining in patients with (n=9) and without (n=11) lymph node metastasis (p=0.2947; Mann-Whitney test). (G) Number of unique TRB sequences in tumor-reactive blood-derived CD8 T cell lines in patients with (n=10) and without (n=11) lymph node metastasis (p=0.1971; Mann-Whitney test). (H) TRB cluster analysis of blood tumor-reactive CD8 T cells (dark blue) and TILs (yellow) using the GLIPH2 algorithm. The link highlights TCRs that are similar based on global alignment of the CDR3β. (I) Graph showing the percentage of TRB sequences that are similar, based on the GLIPH2 analysis, or identical to tumor-reactive TRBs, in patients with (n=5) and without (n=10) lymph node metastasis (p=0.0193; Mann-Whitney test). Related to Figure 4.

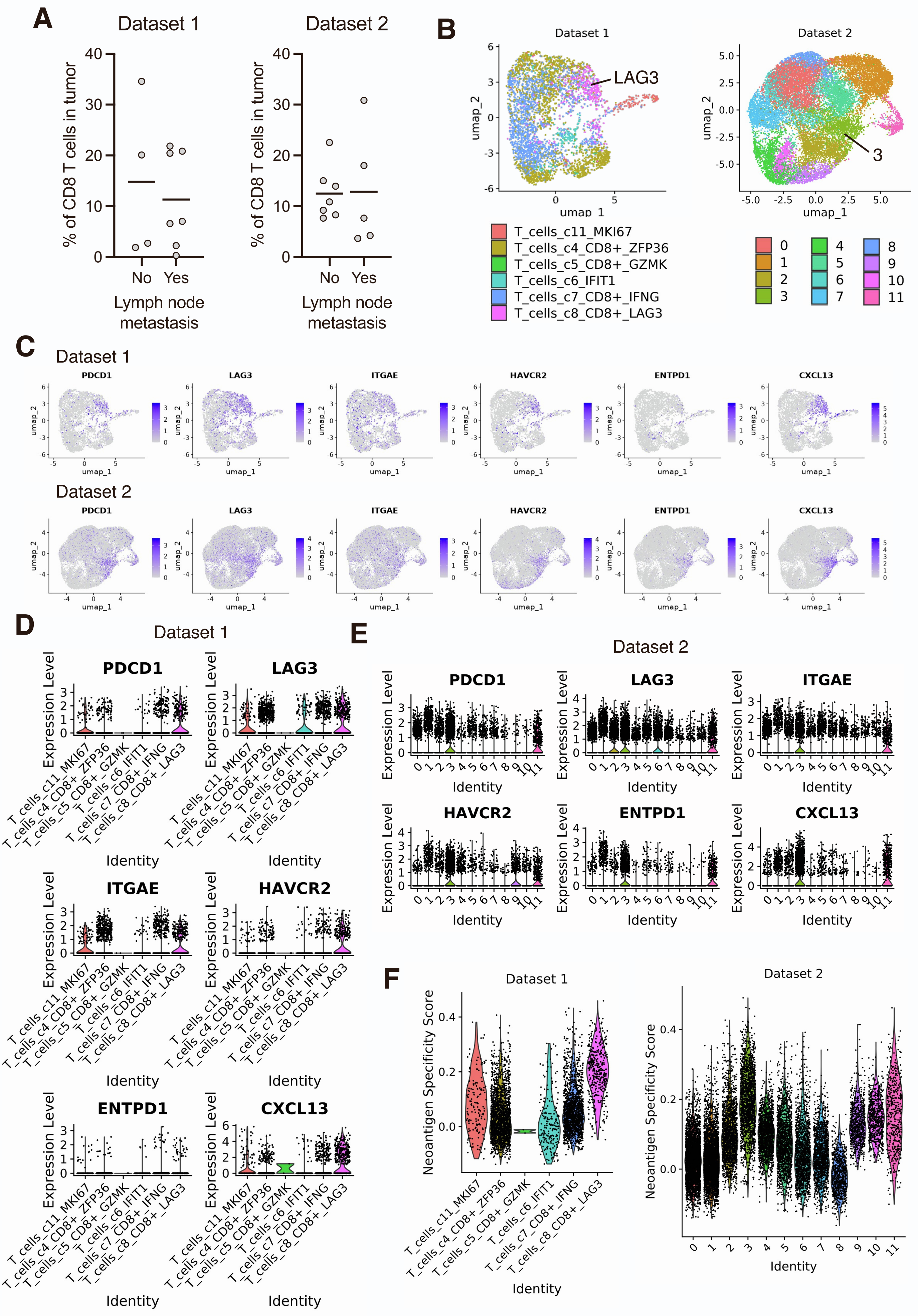
A
Dataset 1
Dataset 2
% of CD8 T cells in tumor
No
Yes
Lymph node metastasis
B
LAG3
3
umap_1
umap_2
T_cells_c11_MKI67
T_cells_c4_CD8+_ZFP36
T_cells_c5_CD8+_GZMK
T_cells_c6_IFIT1
T_cells_c7_CD8+_IFNG
T_cells_c8_CD8+_LAG3
C
PDCD1
LAG3
ITGAE
HAVCR2
ENTPD1
CXCL13
D
E
Expression Level
Identity
F
Neoantigen Specificity Score

Figure S7: Analysis of two independent scRNA-seq datasets. (A) Graph showing the lack of difference in the percentage of CD8 T cells in the tumor of patients with (Dataset 1: n=7; Dataset 2: n=5) and without (Dataset 1: n=4; Dataset 2: n=7) a detected blood tumor-reactive T cell response (Dataset 1: p=0.6482; Dataset 2: p=0.9391; two-tailed unpaired t-test). (B) UMAP plot of scRNA-seq from the two datasets colored by cluster ID. (C) UMAP plots of the two datasets colored by RNA expression of six different genes that correlates with neoantigen specificity. (D) Violin plots of dataset 1 showing the expression of six genes divided by cluster. (E) Violin plots of dataset 2 showing the expression of six genes divided by cluster. (F) Violin plots of dataset 1 and 2 showing the neoantigen specificity score divided by cluster. Related to Figure 5.
